# Calcitonin Gene-Related Peptide (CGRP): Biology, Signaling, Pathophysiological Roles, and Therapeutic Applications

**DOI:** 10.3390/ijms27114973

**Published:** 2026-05-30

**Authors:** María Jesús Ramírez-Expósito, Cristina Cueto-Ureña, José Manuel Martínez-Martos

**Affiliations:** Experimental and Clinical Physiopathology Research Group CTS-1039, Department of Health Sciences, School of Health Sciences, University of Jaén, E23071 Jaén, Spain; mramirez@ujaen.es (M.J.R.-E.); ccueto@ujaen.es (C.C.-U.)

**Keywords:** CGRP, neuropeptide, migraine, gepants, monoclonal antibodies, neurogenic inflammation, pain, CLR-RAMP1 receptor

## Abstract

The calcitonin gene-related peptide (CGRP) is a 37-amino acid neuropeptide belonging to the calcitonin family, discovered as a product of alternative splicing of the calcitonin gene. CGRP has emerged as a pleiotropic signaling molecule with widespread distribution in the central and peripheral nervous systems, particularly within primary sensory neurons. This narrative review synthesizes current knowledge on the CGRP system, integrating recent advances in its molecular structure, gene organization, and post-translational processing with high-resolution structural insights into its heterodimeric receptor complex (CLR-RAMP1) obtained through cryo-electron microscopy. We also include long-term safety data on anti-CGRP monoclonal antibodies, emerging cardiovascular risk signals, and novel therapeutic applications in vestibular migraine and pediatric populations. The intracellular signaling cascades activated by CGRP, including the canonical cAMP-PKA pathway, MAP kinase activation, and context-dependent calcium signaling, are discussed in relation to its diverse physiological functions. These encompass vasodilation, nociception modulation, neurogenic inflammation, gastrointestinal motility, bone metabolism, tissue regeneration, and energy homeostasis. The central role of CGRP in migraine pathophysiology is examined to understand the development of targeted therapies. The current pharmacological landscape is reviewed, including the evolution of small-molecule CGRP receptor antagonists (gepants) through three generations and the four approved monoclonal antibodies targeting CGRP or its receptor, with comparative analysis of their efficacy, safety profiles, and clinical positioning. Beyond migraine, emerging and predominantly preclinical roles of the CGRP system are discussed in chronic pain, osteoarthritis, cardiovascular diseases, sepsis, cancer (particularly bone metastases and tumor microenvironment immunomodulation), and neurodegenerative disorders such as Alzheimer’s disease. In these areas, the available evidence remains heterogeneous and, in most cases, is not yet sufficient to support clinical translation. Finally, future directions are discussed, including the development of stable CGRP analogs, allosteric modulators, and the potential expansion of therapeutic applications into oncology, intensive care medicine, and neuroprotection.

## 1. Introduction

Calcitonin gene-related peptide (CGRP) represents one of the most widely distributed neuropeptides in the mammalian nervous system with diverse functions, and its clinical relevance has been increasingly recognized over the past four decades [1]. The discovery of CGRP and the proposal of its generation via alternative splicing was first described by Amara and colleagues in 1982 [2], with subsequent structural and tissue characterization in 1983 by Rosenfeld and colleagues [3]. CGRP emerged from the study of alternative processing of the calcitonin gene, and showed that a single gene could generate distinct protein products depending on the cell type through alternative splicing mechanisms of messenger RNA [4,5]. This finding expanded the understanding of gene processing in eukaryotes and introduced a new peptide whose biological functions proved to be broad and whose pharmacological modulation would eventually acquire therapeutic importance in the treatment of migraine [6,7].

CGRP is predominantly expressed in small- and medium-diameter primary sensory neurons of the dorsal root ganglia and the trigeminal ganglion, acting as a bidirectional mediator that couples peripheral tissue responses with central nociceptive processing [1,5,8]. Its pleiotropic biological functions encompass vasodilation, nociception, neurogenic inflammation, bone metabolism, gastrointestinal motility, and energy homeostasis [1,5,8,9,10].

The pathophysiological importance of CGRP has been most clearly established in migraine, where elevated cranial CGRP levels during attacks, the capacity of exogenous CGRP to trigger migraine in susceptible individuals, and the therapeutic efficacy of gepants and monoclonal antibodies collectively validate this neuropeptide as a central mediator of migraine pain [5,6,7,11]. The development of these agents exemplifies rational drug design grounded in molecular pathophysiology and has transformed the clinical management of migraine [11,12]. Beyond migraine, CGRP involvement in chronic pain, osteoarthritis, bone metastases, cardiovascular diseases, sepsis, and neurodegenerative disorders positions this system as a therapeutic target across a broader disease spectrum [12,13,14].

The aim of this narrative review is to provide an update on current knowledge about CGRP, integrating aspects of its molecular structure, its gene and post-translational processing, the organization of the receptor complex, intracellular signaling cascades, its physiological functions in different organ systems, its involvement in pathophysiological processes, and its current and potential therapeutic applications. We pay special attention to recent advances in the structural characterization of the CGRP receptor by cryo-electron microscopy [15], to the identification of new physiological roles of the peptide in processes such as wound healing and energy metabolism [16], and to the development of pharmacological strategies targeting the CGRP system that have changed the clinical management of migraine [12,15,17,18]. Furthermore, we integrate key findings related to real-world long-term effectiveness [19,20], safety signals from pharmacovigilance databases [21,22], and the emerging role of CGRP in new disease contexts such as cochlear implantation [23] and Parkinson’s disease [24]. An integrated understanding of CGRP biology, from the molecular to the clinical perspective, provides insight into neuropeptide signaling mechanisms and also serves as a reference for the development of targeted therapies based on the molecular pathophysiology of neurological and systemic diseases. In addition, this review seeks to bridge mechanistic and clinical perspectives while distinguishing between areas supported by established clinical evidence and those that remain preclinical, exploratory, or controversial. We also highlight areas in which knowledge remains incomplete or where translational development is still at an early stage [12,25].

## 2. Gene Structure and Processing

CGRP exists in two isoforms, designated CGRP-α and CGRP-β, which are encoded by different genes located on human chromosome 11 [1,26]. The *CALCA* gene, also known as *CT/CALCA*, generates CGRP-α through an alternative RNA processing mechanism that allows it to produce both calcitonin and CGRP-α depending on the cell type. The *CALCB* gene, in turn, exclusively encodes CGRP-β and does not generate calcitonin, with a predominant expression pattern in the enteric nervous system and internal organs [1].

The structure of the *CALCA* gene consists of six exons that allow the generation of alternative transcripts through the selective use of polyadenylation sites specific for calcitonin or for CGRP [27]. In thyroid C cells, processing of the primary transcript includes exon 4, resulting in the production of calcitonin messenger RNA [27,28]. In contrast, in neurons of the central and peripheral nervous system, processing includes exons 5 and 6, while exon 4 is excluded, thus generating CGRP-α messenger RNA [1,27]. This alternative processing mechanism is regulated by tissue-specific differences in the components of the constitutive splicing machinery and the specific regulatory protein SRp55. SRp55 acts as a key regulator by binding to an exonic splice enhancer (ESE) located within exon 4; its presence in cells like thyroid C cells promotes the inclusion of exon 4 to produce calcitonin, whereas its lower levels in neurons favor the exclusion of this exon to generate CGRP-α mRNA [28]. The common region of both transcripts includes exons 1, 2, and 3, which encode the signal peptide and shared regulatory regions [1].

The *CALCB* gene has a similar organization with five exons, where exon 1 is untranslated, exon 2 encodes the signal peptide, and exon 3 contains the sequence encoding the mature peptide. The two CGRP isoforms share more than 90% homology and differ in only three amino acids in their 37-residue sequence, explaining why they share similar biological activities [1,26]. The tissue distribution of the two isoforms is different, with CGRP-α being predominant in the central and peripheral nervous system, whereas CGRP-β is expressed mainly in the enteric nervous system [1].

Post-translational processing of CGRP begins with translation of the messenger RNA into a 121-amino acid prohormone (proCGRP), which must undergo several enzymatic modifications to generate the mature 37-amino acid peptide [1]. The first step is endoproteolytic cleavage at tetrabasic sites, mediated by the prohormone convertases PC1 and PC2 [29]. These enzymes belong to the neuroendocrine endoprotease family and are responsible for processing multiple peptide prohormones by recognizing and cleaving at pairs of basic amino acids. In the case of CGRP, PC1 has been shown to play a major role in processing proCGRP, whereas procalcitonin requires the combined action of PC2 and PC1 [29].

Following endoproteolytic cleavage, the peptide undergoes removal of the basic residues constituting the cleavage site, followed by other modifications necessary for the biological activity of mature CGRP. The sequential action of prohormone convertases, carboxypeptidases, and amidating enzymes thus generates the mature 37-amino acid peptide, whose critical structural features include the N-terminal disulfide ring between cysteines 2 and 7 and the C-terminal phenylalanine amide group, both essential for receptor binding and biological activity [1,30].

The mature peptide is stored in dense-core vesicles within sensory nerve terminals, where it remains until release through calcium-dependent exocytosis [31]. This release process is mediated by members of the SNARE protein family, which are classic components of exocytic pathways [32]. Once released into the extracellular space, CGRP can be degraded by metalloproteases, with insulin-degrading enzyme (IDE) being especially important in the proteolytic processing of the peptide at specific cleavage sites [33].

## 3. Primary and Secondary Structure

CGRP is a 37-amino acid neuropeptide belonging to the calcitonin family, which also includes amylin, adrenomedullin, and calcitonin itself [5,9,13,14]. The primary structure of human CGRP-α is conserved across different species, with 23 of the 37 residues being identical in all vertebrates studied [5,14]. Differences between human and rodent species are limited to four amino acid positions located at positions 1, 3, 25, and 35, although these substitutions do not significantly alter the biological activity of the peptide [14]. Comparison between the human isoforms CGRP-α and CGRP-β shows that they share more than 90% homology and differ in only three amino acids located at positions 3, 22, and 25, with position 3 being aspartic acid in CGRP-α and asparagine in CGRP-β [5,9,13,14].

The primary structure of CGRP has two post-translational features necessary for its biological activity that are conserved in all members of the calcitonin family. The first is the presence of an intramolecular disulfide bond between cysteine residues at positions 2 and 7, which generates a six-amino acid ring at the amino terminus [9,13,14]. This disulfide bridge is necessary for peptide function, as its removal or modification by carboxamidomethylation markedly reduces affinity and almost completely eliminates biological activity [9,14]. The second modification is the amidation of the phenylalanine residue at position 37 of the carboxy terminus, resulting in the formation of a phenylalanine-amide group [5,9,14]. This C-terminal amidation is necessary for peptide binding to the extracellular domain of its receptor and for subsequent activation [14].

Structural studies using nuclear magnetic resonance spectroscopy and circular dichroism have shown that CGRP does not have a rigid secondary structure in aqueous solution but rather adopts mostly a random coil conformation under these conditions [5,34]. However, addition of solvents that favor secondary structure formation, such as hexafluoro-2-propanol, induces a conformational transition that stabilizes regions with α-helical structure [5,34]. This ability to adopt more structured conformations in hydrophobic environments suggests that the peptide likely acquires a more defined structure when interacting with lipid membranes or with its receptor [5,34].

The secondary structure of CGRP is organized into four functional domains that have been characterized through structure-activity relationship studies and spectroscopic techniques [9,13]. Domain 1 comprises residues 1 through 7 and forms a ring structure stabilized by the disulfide bond between cysteines 2 and 7 [9,13,14]. This region is necessary for receptor activation, since the truncated fragment CGRP 8–37, which lacks these residues, acts as a competitive antagonist with approximately ten-fold lower affinity than the full-length peptide but with no agonist activity [1,5,9]. The N-terminal ring interacts with the transmembrane domain and extracellular loops of the receptor, triggering the conformational changes necessary for activation [5,9].

Domain 2 extends from residue 8 to 18 and constitutes the region with the highest propensity to form an amphipathic α-helix [9,13,14]. Structural studies indicate that this helix is stabilized by hydrophobic interactions and hydrogen bonds, particularly those involving the threonine residue at position 9 [1,13]. The sequence of this region shows a conserved pattern of hydrophobic residues, especially leucines at positions 12 and 16, which appear to occupy one face of the helix and establish steric contacts with the receptor [14]. Deletion of this helical region reduces the affinity of the peptide for its receptor by 50- to 100-fold, demonstrating its importance for high-affinity binding [9,13,14].

Domain 3 spans residues 19 to 27 and is characterized by the presence of a β- or γ-turn that introduces flexibility into the peptide structure [9,13,14]. The serine-glycine-glycine sequence at positions 19–21 is conserved across species and is responsible for generating this structural turn [9]. Replacement studies with β-turn dipeptides at these positions have shown that introducing such analogs maintains normal peptide affinity, confirming the functional importance of this turn for the bioactive conformation [9,13].

Domain 4 comprises the final residues 28 through 37 and constitutes the C-terminal region that initially interacts with the extracellular domain of the receptor according to the two-domain model proposed for family B G protein-coupled receptors [1,5,34]. Nuclear magnetic resonance studies have detected the presence of two turn regions in this zone, specifically between residues 28–30 and 33–34, which generate a multiply folded structure [9,13]. The amidated phenylalanine at position 37 is especially important, as its removal or substitution with other amino acids results in losses of affinity greater than 100-fold [9,13,14]. Other important residues in this domain include proline at position 29 and threonine at position 30, whose substitution with alanine completely eliminates receptor binding ability [34].

## 4. Stability and Kinetics

CGRP has a short plasma half-life that limits its duration of action in vivo and determines its function as a rapid modulator of physiological responses. Pharmacokinetic studies have shown that endogenous CGRP has a half-life of approximately five minutes in human plasma, with a biphasic elimination kinetics characterized by a first phase of 6.9 min and a second phase of 26.4 min [11]. However, the development of acylated CGRP analogs, such as SAX, has achieved an extended half-life of approximately 7 h [9,35]. These stable analogs have demonstrated efficacy in reducing blood pressure and protecting against heart failure in murine models. More recently, computational design and molecular dynamics simulations have enabled the development of stapled peptide-based antagonists with enhanced serum stability and retained receptor antagonism [36]. This rapid plasma elimination of the native peptide is consistent with experimental observations showing complete reversal of the vascular effects of the peptide within 20 min after its administration [1], whereas acylated versions allow for prolonged therapeutic effects on the cardiovascular and nociceptive systems [35]. The short half-life of CGRP is compatible with its physiological role as a neuropeptide released locally in response to specific stimuli, allowing precise temporal regulation of its effects on the cardiovascular and nociceptive systems [1].

Proteolytic degradation constitutes the main mechanism responsible for the elimination of circulating CGRP and the termination of its biological action. IDE has been identified as the primary candidate responsible for CGRP degradation through proteomic studies combined with biochemical enzyme fractionation assays [5]. Experiments with purified recombinant enzymes demonstrated that IDE is capable of cleaving CGRP at two specific sites, generating the fragments CGRP 1–17 and CGRP 18–37 by cleavage between serine 17 and arginine 18, as well as fragments CGRP 1–26 and CGRP 27–37 by cleavage between asparagine 26 and phenylalanine 27 [5]. The physiological relevance of IDE in CGRP metabolism has been confirmed through studies in mice deficient in this enzyme, which exhibit elevated levels of intact CGRP in both spinal cord tissue lysates and plasma [5].

Neprilysin, also known as enkephalinase or neutral endopeptidase, represents another metalloproteinase involved in CGRP degradation, although its relative contribution appears to be less than that of IDE in most tissues [1]. Studies performed in human skin have shown that neprilysin, but not angiotensin-converting enzyme (ACE), participates in cutaneous CGRP degradation, and that neprilysin inhibitors facilitate neurogenic inflammation mediated by this neuropeptide [1]. The activity of neprilysin on CGRP is consistent with its known substrate specificity, which includes preferential cleavage on the amino side of hydrophobic residues [5]. However, the contribution of neprilysin to systemic degradation of CGRP appears to be limited, as evidenced by the fact that selective neprilysin inhibitors do not produce substantial increases in circulating CGRP levels under physiological conditions [1].

Endothelin-converting enzyme-1 (ECE-1) has also been implicated in CGRP degradation, particularly in specific pathological contexts [1]. Studies suggest that ECE-1 may contribute to local regulation of CGRP levels in certain vascular beds and in the lung, where this enzyme is abundantly expressed [5]. The functional relevance of CGRP degradation by ECE-1 has been demonstrated in murine models of pulmonary fibrosis, where modulation of this enzyme’s activity affects disease progression [1,5]. Nevertheless, the relative contribution of ECE-1 versus IDE to total systemic CGRP degradation remains an area requiring further investigation to establish the quantitative importance of each proteolytic pathway in different physiological and pathological contexts [5].

Mass spectrometry analyses of peptides incubated with human or primate plasma have also enabled the development of analogs with increased stability [5]. The antagonist fragment CGRP 8–37, which lacks the N-terminal ring necessary for receptor activation, has limited plasma stability with a half-life of approximately one hour in cynomolgus monkey plasma [5]. Studies aimed at identifying specific proteolytic degradation sites have revealed that positions 11, 18, 24, 29, and 34 are particularly susceptible to attack by plasma proteases. Iterative substitution of amino acids at these positions with unnatural residues such as citrulline, homoarginine, 2-naphthylalanine, and octahydroindole-2-carboxylic acid has allowed the generation of analogs with increased stability of more than 100-fold, achieving half-lives of 38 h in cynomolgus monkey plasma and 68 h in human plasma [5].

The binding kinetics of CGRP to its receptor constitute another aspect of its pharmacological profile that determines the duration of cellular signaling. Recent studies employing surface plasmon resonance techniques and functional assays have revealed that CGRP exhibits relatively rapid dissociation kinetics from the calcitonin receptor-like receptor/receptor activity-modifying protein 1 complex, with a receptor residence time of approximately 13 min [5]. This receptor binding kinetics, combined with rapid plasma degradation, explains the transient nature of CGRP-mediated physiological responses. The molecular determinants of the peptide’s residence time on the receptor have been investigated using designed analogs, demonstrating that specific modifications at positions 14, 17, 19, 20, and 25 can alter both binding affinity and dissociation kinetics [5]. Lipidated analogs of the CGRP 8–37 fragment conjugated with long-chain fatty acids at these positions have shown extended plasma half-lives ranging from 7.3 to 13.7 h in mice, representing an increase of more than 100-fold compared to the unmodified peptide [5].

## 5. CGRP Receptor Complex and Molecular Pharmacology

**Figure 1:**
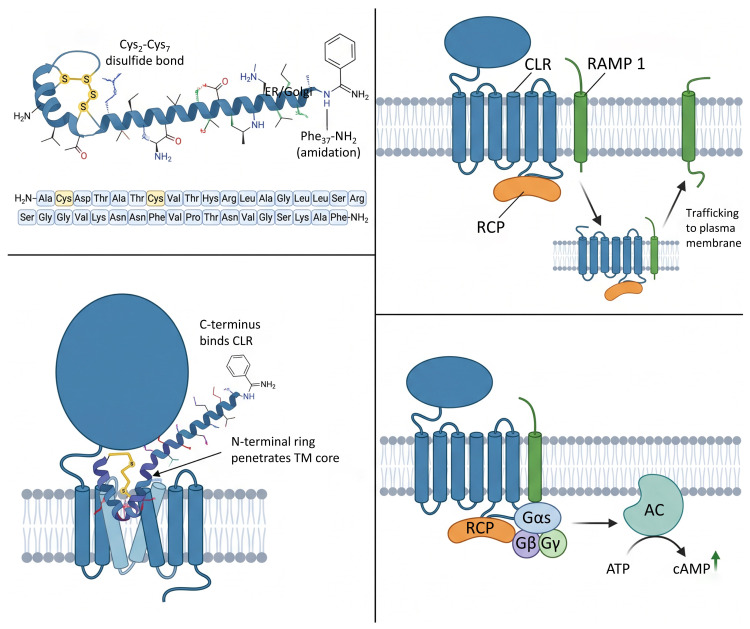
Molecular organization of CGRP and its functional receptor. CGRP is a 37-amino acid neuropeptide whose biological activity requires two critical post-translational modifications: an intramolecular disulfide bond between cysteine residues at positions 2 and 7, forming a six-amino acid N-terminal ring, and amidation of the C-terminal phenylalanine. The canonical CGRP receptor is a heterotrimeric complex composed of the calcitonin receptor-like receptor (CLR, a seven-transmembrane domain protein belonging to class B1 GPCRs), receptor activity-modifying protein 1 (RAMP1, a single-pass transmembrane protein), and the receptor component protein (RCP, an intracellular peripheral membrane protein essential for Gs coupling). RAMP1 is indispensable for CLR trafficking to the plasma membrane and confers selectivity for CGRP over other calcitonin family peptides. CGRP binding follows a two-domain model: the peptide C-terminus initially engages the CLR extracellular domain, while the N-terminal ring penetrates the transmembrane core, contacting extracellular loops and transmembrane helices. This interaction stabilizes an active receptor conformation that promotes Gs coupling and activation, the main downstream signaling pathway.

The CGRP receptor represents a heterotrimeric system formed by the association of three distinct protein components, the calcitonin receptor-like receptor (CLR), receptor activity-modifying protein 1 (RAMP1), and the receptor component protein (RCP) [5,14,37,38]. While CLR and RAMP1 dictate ligand binding at the cell surface, RCP is an essential intracellular peripheral membrane protein required to couple the CLR/RAMP1 complex to the Gs protein, thereby enabling downstream signal transduction and receptor functionality [5,14,15,37]. The calcitonin receptor-like receptor belongs to the B1 family of G protein-coupled receptors and features seven characteristic transmembrane domains, preceded by an extracellular amino-terminal domain of approximately 140 amino acids [5,14]. In the absence of RAMP1, CLR does not reach the plasma membrane and remains retained in the endoplasmic reticulum, demonstrating that association with RAMP1 is essential not only for determining ligand selectivity but also for proper intracellular trafficking and functional expression of the receptor [5,11]. The stoichiometry of the complex has been confirmed by cryo-electron microscopy structural studies revealing an organization in which RAMP1 establishes specific contacts with the transmembrane and extracellular domains of CLR [5,15] (Figure 1).

RAMP1 is a single-pass membrane protein with an extracellular domain of approximately 100 amino acids, a transmembrane domain, and a short intracellular domain of fewer than 10 amino acids [5,13,14]. The role of RAMP1 in determining receptor selectivity is clear, as the same CLR protein can function as a CGRP receptor when associated with RAMP1, or as an adrenomedullin receptor when associated with RAMP2 or RAMP3 [5,9,13,14]. Structural studies have revealed that RAMP1 establishes extensive contacts with the extracellular domain of CLR, stabilizing a conformation that favors high-affinity CGRP binding [5,15]. The transmembrane domain of RAMP1 is positioned at the interface between transmembrane domains 3, 4, and 5 of CLR, where it contributes to stabilizing the extracellular loop 2 of the receptor, an important region for peptide binding [15].

Cryo-electron microscopy studies of the CGRP-CLR-RAMP1-Gs protein complex have provided detailed structural information on the receptor activation mechanism, with a resolution of 3.3 angstroms [5,15]. The CGRP establishes extensive interactions with the receptor complex, with approximately 61.5% of its total surface buried in the binding interface. The carboxy terminus of the peptide, particularly the amidated phenylalanine at position 37, initially binds to the extracellular domain of CLR in a molecular recognition mode characteristic of class B1 receptors [13,15]. This initial contact allows the amino terminus of the peptide to penetrate into the core of the transmembrane domain, where the ring formed by the disulfide bond between cysteines 2 and 7 establishes contacts with the extracellular loops and transmembrane domains of the receptor [5].

Activation of the CGRP receptor triggers the coupling and activation of the heterotrimeric Gs protein, which constitutes the main signal transducer for this receptor [5,13,14]. The structure of the active complex reveals that Gs establishes extensive contacts with the intracellular regions of CLR, particularly with the intracellular portion of transmembrane domains 5 and 6, as well as with intracellular loops 2 and 3 [5,39]. Molecular dynamics studies indicate that RAMP1 provides additional stability to the receptor-G protein complex, especially in the region of the extracellular domain of CLR, thereby facilitating proper presentation of the peptide to the receptor core and subsequent G protein activation [5,39]. Comparative analysis with structures of the calcitonin receptor suggests that the inward movement of the apex of transmembrane domain 5 of CLR by approximately 2 angstroms is a direct consequence of interaction with RAMP1 and represents an important structural determinant for ligand selectivity [5].

The primary intracellular consequence of CGRP receptor activation is Gs-mediated stimulation of adenylyl cyclase, with the resulting cAMP elevation and PKA activation constituting the canonical pathway through which CGRP exerts its vasodilatory, nociceptive, and gene-regulatory effects [5,14].

The structural characterization of the CLR-RAMP1 complex has provided the molecular framework for the rational design of CGRP receptor antagonists. Gepants bind to a cavity formed by the extracellular domain of CLR and the transmembrane domains, partially occupying the same space as the carboxy terminus of the CGRP, and their antagonist activity is RAMP-dependent, with differences in inhibitory potency depending on the RAMP isoform associated with CLR [5]. Kinetic studies using enhanced molecular simulations have further elucidated the determinants of improved CGRP peptide binding kinetics, revealing that engineered variants such as ssCGRP exhibit longer residence times due to enhanced ligand recapture and a more heterogeneous bound-state ensemble [40].

## 6. Molecular Signaling Pathways

**Figure 2:**
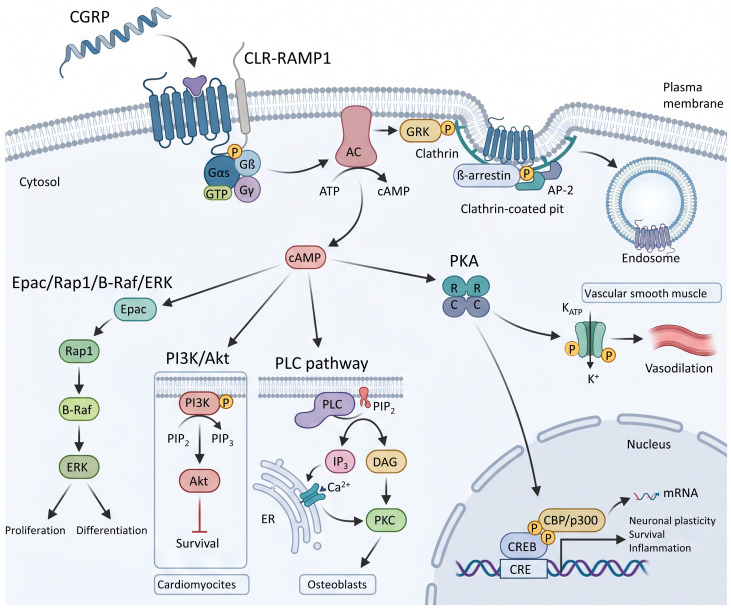
Intracellular signaling cascades triggered by CGRP receptor activation. Upon CGRP binding, the CLR-RAMP1 complex undergoes a conformational change that promotes coupling and activation of the heterotrimeric G protein Gs. The dissociated Gαs subunit stimulates adenylyl cyclase (AC), increasing intracellular cAMP concentration, which in turn activates protein kinase A (PKA). PKA phosphorylates multiple substrates, including ATP-sensitive potassium (K_ATP_) channels in vascular smooth muscle (a key mechanism underlying CGRP-induced vasodilation) and the transcription factor CREB (cAMP response element-binding protein), which regulates gene expression involved in neuronal plasticity, survival, and inflammation. Beyond the cAMP-PKA axis, CGRP engages additional pathways in a cell-type-specific manner. The Epac/Rap1/B-Raf/ERK cascade is involved in proliferation and differentiation; the PI3K/Akt pathway, exerts in cardiomyocytes a net inhibitory effect on survival, contrasting with other cardioprotective neuropeptides; the phospholipase C (PLC) pathway, which hydrolyzes phosphatidylinositol 4,5-bisphosphate (PIP_2_) to generate inositol trisphosphate (IP_3_) and diacylglycerol (DAG), mobilizes calcium from intracellular stores and activates protein kinase C (PKC), a pathway particularly relevant in osteoblasts. Sustained receptor activation triggers GRK-mediated phosphorylation, β-arrestin recruitment, and clathrin-dependent internalization, mechanisms that limit the duration and intensity of CGRP signaling.

Activation of the CGRP receptor triggers multiple intracellular signaling cascades (Figure 2) that converge to produce the physiological effects of the peptide, with the cyclic adenosine monophosphate pathway being the predominant route in most cell types [5,9,13,14,35]. Coupling of the activated receptor to the heterotrimeric Gs protein results in dissociation of the Gαs subunit, which binds and activates adenylyl cyclase to catalyze the conversion of ATP to cAMP [8,9,13,35]. The increase in intracellular cAMP levels occurs within the first seconds after CGRP stimulation and reaches concentrations that can be 10- to 20-fold higher than basal levels, depending on the cell type and receptor density [13,41]. cAMP accumulation activates protein kinase A through binding of four cAMP molecules to the two regulatory subunits of the inactive holoenzyme, causing release of the two active catalytic subunits [13,41]. The catalytic subunits of PKA phosphorylate multiple cellular substrates on serine or threonine residues found in specific sequence contexts, modifying the activity of metabolic enzymes, ion channels, cytoskeletal proteins, and transcription factors [5,8,13].

One of the PKA targets in vascular smooth muscle cells is ATP-sensitive potassium channels (K_ATP_), whose activation by CGRP importantly contributes to the vasodilatory effects of the peptide [5,9,13,14,41]. Electrophysiological studies in porcine coronary artery smooth muscle cells have demonstrated that CGRP activates potassium currents that are blocked by glibenclamide, a specific inhibitor of K_ATP_ channels [13,41]. The magnitude of the CGRP-activated current depends on the intracellular ATP concentration, being larger when cells are dialyzed with 0.1 millimolar ATP compared to 3.0 millimolar, which is consistent with negative regulation of these channels by intracellular ATP [13,41]. The mechanism by which PKA activates K_ATP_ channels involves direct phosphorylation of channel regulatory subunits, particularly the sulfonylurea receptor subunits, which increases the channel opening probability [13,41]. The competitive PKA inhibitor Rp-cAMPS reduces the CGRP-activated current by approximately 60% without affecting currents activated by direct K_ATP_ channel openers such as pinacidil, confirming that PKA mediates this effect [13,41].

Activation of mitogen-activated protein kinases (MAPK) constitutes another signaling pathway triggered by CGRP, with distinct temporal kinetics and specific cellular functions [13,42,43]. CGRP stimulates the phosphorylation and activation of ERK1/2 through mechanisms involving both the cAMP-PKA pathway and synergistic interactions with calcium signals [5,13,17,42,43]. In Schwann cells, activation of the CGRP receptor induces a proinflammatory response by stimulating the production of IL-1β and IL-6 via the cAMP-PKA-ERK cascade, suggesting that the peptide plays a direct role in the initiation of inflammatory processes in the peripheral nervous system [42]. Studies in human osteosarcoma cell lines expressing CGRP receptors have shown that the peptide induces a rapid increase in ERK1/2 phosphorylation that peaks between 5 and 15 min after stimulation [42]. The molecular mechanism by which PKA activates ERK involves phosphorylation of Src family proteins, which are non-receptor tyrosine kinases that act as intermediaries between G protein-coupled receptors and the Raf-MEK-ERK cascade [44]. PKA phosphorylates Src at specific residues that modulate its catalytic activity, allowing phosphorylation of tyrosines on adaptor proteins that recruit and activate Raf, the initial kinase of the MAPK cascade. In addition, PKA can activate ERK through Epac, a guanine nucleotide exchange factor for Rap1 that is directly activated by cAMP independently of PKA and leads to B-Raf activation [17,43].

The transcription factor CREB represents a nuclear effector of CGRP-activated pathways that mediates changes in gene expression related to cellular plasticity, survival, and differentiation [5,8,13]. CREB is phosphorylated at serine 133 by both PKA and ERK, and this phosphorylation is necessary for recruitment of transcriptional coactivators such as CBP and p300. Studies have shown that CGRP activation leads to sustained CREB phosphorylation, which can persist for hours after initial stimulation, reflecting prolonged ERK activation rather than transient PKA activation [5]. Phosphorylation of CREB by both kinases represents a point of convergence of the cAMP and MAPK pathways, allowing integration of signals that results in transcriptional activation levels higher than those achieved by each pathway alone [43]. Genes regulated by CREB in response to CGRP include trophic factors, neuropeptides, metabolic enzymes, and anti-apoptotic proteins that contribute to the neuroprotective and cardioprotective effects of the peptide [13].

The phosphatidylinositol 3-kinase (PI3K) and its effector Akt pathway represent another signaling cascade modulated by CGRP, although its regulation shows complexity and contextual specificity [13,17,45]. Studies in cardiomyocytes have revealed that CGRP can exert inhibitory effects on Akt activation, in contrast to the activating effects observed with other cardioprotective neuropeptides [13,45]. CGRP treatment produces a reduction in Akt gene expression, as well as a decrease in Akt phosphorylation levels at threonine 308 and serine 473, which are markers of its full activation [13,45]. This suppression of the PI3K/Akt pathway by CGRP is accompanied by decreases in expression of Akt-dependent anti-apoptotic genes such as nuclear factor κB (NFκB) and superoxide dismutase 3 (SOD3), suggesting negative modulation of cell survival pathways [13,45]. In parallel, this CGRP-induced inhibition of Akt results in a compensatory increase in p38 MAPK protein expression, possibly due to release from the inhibition that Akt normally exerts on p38 synthesis [13,45]. The CGRP receptor antagonist 8–37 prevents all these effects, confirming that signaling is mediated by the CLR-RAMP1 receptor and not by receptor-independent mechanisms [13].

The interaction between CGRP-mediated signaling and nitric oxide production represents an aspect of the vascular physiology of the peptide, although the nature of this interaction varies according to the vascular bed and temporal context [5,9,46]. In endothelial cells, CGRP can stimulate nitric oxide production through activation of endothelial nitric oxide synthase, contributing to vasodilation in certain vascular territories [5,9,13,46]. The mechanism involves phosphorylation of eNOS at serine 1177 by kinases activated downstream of the CGRP receptor, particularly Akt and PKA, which increases the catalytic activity of the enzyme [5,9,46]. However, recent studies have revealed that prolonged CGRP stimulation can inhibit nitric oxide production through opening of pannexin-1 channels and subsequent activation of connexin-based hemichannels [46]. This channel activation leads to NADPH oxidase-dependent superoxide anion generation, which reacts with nitric oxide to form peroxynitrite, reducing nitric oxide bioavailability and producing endothelial dysfunction [46]. The development of this CGRP-induced endothelial dysfunction is time-dependent, requires more than one hour of continuous stimulation, and can be prevented by pannexin-1 inhibitors or by superoxide radical scavengers such as TEMPOL [46].

Phospholipase C (PLC) represents an additional signaling pathway activated by CGRP in certain cell types, particularly in bone cells and in some smooth muscle cell populations [13,43,47,48]. In human OHS-4 bone cells expressing CGRP receptors, stimulation with the peptide selectively activates the PLC pathway without producing significant cAMP accumulation [48]. Activation of PLC-β1 by CGRP in these cells is mediated by coupling of the receptor to Gαq/11 proteins, representing an example of coupling promiscuity of the CLR-RAMP1 receptor that can vary according to cellular context and expression levels of different G proteins [13,43,47,48]. PLC activation results in hydrolysis of phosphatidylinositol 4,5-bisphosphate to generate inositol 1,4,5-trisphosphate and diacylglycerol, which mobilize calcium from intracellular stores and activate protein kinase C isoforms, respectively [13,48]. The increase in intracellular calcium concentration induced by CGRP in these cells occurs within 5 s and results both from calcium influx through voltage-dependent calcium channels and from calcium release from the endoplasmic reticulum [13,48]. Recent evidence has identified the transient receptor potential melastatin 3 (TRPM3) channel as a novel activator of CGRP release in trigeminal neurons. TRPM3 activation with the agonist CIM0216 triggers CGRP release from trigeminal ganglia and induces vasodilation, implicating TRPM3 as a potential therapeutic target in migraine [49].

Desensitization of the CGRP receptor and its internalization constitute essential regulatory mechanisms that limit the duration and magnitude of peptide signaling [5,11,50]. Phosphorylation of the activated receptor by G protein-coupled receptor kinases constitutes the initial event in homologous desensitization, creating high-affinity recognition sites for β-arrestins [5,11,14,43,50]. Studies of β-arrestin recruitment to the CLR-RAMP1 complex have revealed that both β-arrestin isoforms, β-arrestin-1 and β-arrestin-2, are recruited to the receptor following CGRP stimulation, albeit with slightly different kinetics [5,43,50]. Binding of β-arrestins to the phosphorylated receptor sterically uncouples the G protein, terminating Gs-dependent signaling, and at the same time initiates receptor internalization by mediating interactions with clathrin and endocytic adaptor proteins [5,11,50]. Gene silencing experiments of β-arrestins by RNA interference have demonstrated that agonist-dependent internalization of the CGRP receptor requires the presence of β-arrestins, as cells deficient in both isoforms show reduced receptor internalization [43,50]. Modulation of the expression of different GRKs, in particular GRK4, GRK5, and GRK6, affects both β-arrestin recruitment and receptor expression at the cell surface, with overexpression of these kinases producing an agonist-independent decrease in surface receptors, likely due to constitutive phosphorylation and internalization [5,50,51].

## 7. Physiology of CGRP

**Figure 3:**
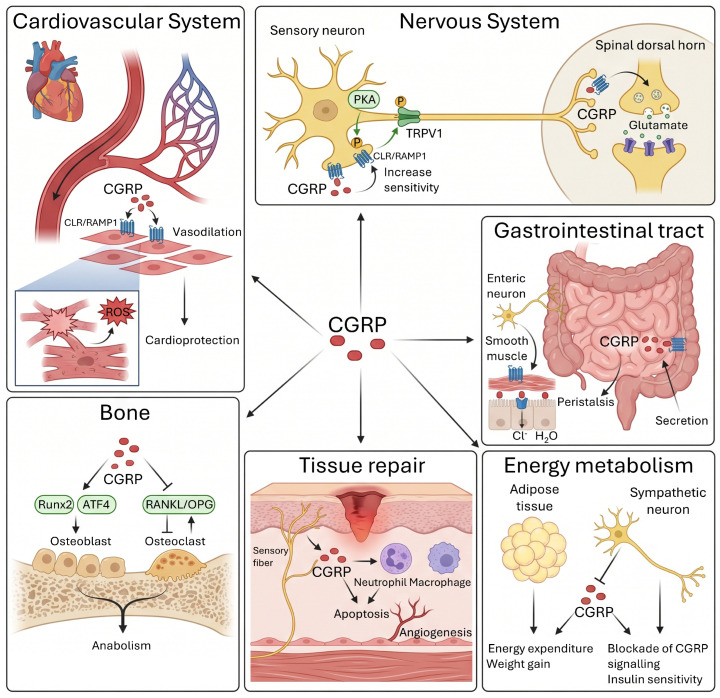
Spectrum of CGRP physiological actions across different organs and systems. CGRP exerts pleiotropic functions beyond its classic role as a vasodilator. In the cardiovascular system, it is one of the most potent endogenous vasodilators and provides protection against myocardial ischemia–reperfusion injury. In the nervous system, it sensitizes peripheral nociceptors via PKA-dependent phosphorylation of TRPV1 channels and facilitates glutamatergic transmission in the spinal dorsal horn, contributing to central sensitization. In the gastrointestinal tract, it stimulates peristaltic motility and ion/water secretion, explaining why constipation is a common adverse effect of CGRP antagonists. In bone, CGRP promotes osteoblastic differentiation (through Runx2 and ATF4) and inhibits osteoclastogenesis by modulating the RANKL/OPG axis, exerting a net anabolic effect that declines with age. In tissue repair, CGRP released from sensory fibers reduces excessive neutrophil and macrophage recruitment, induces their apoptosis, and promotes angiogenesis, thereby accelerating cutaneous wound healing and muscle regeneration. Finally, in energy metabolism, endogenous CGRP contributes to high-fat diet-induced weight gain by inhibiting sympathetic activity and reducing energy expenditure; its blockade improves insulin sensitivity and reduces adiposity in murine obesity models.

The following subsections describe the main physiological actions of CGRP in organ systems in which its role has been most clearly characterized, proceeding from cardiovascular and nociceptive functions to bone metabolism, tissue repair, and energy homeostasis. The main physiological actions of CGRP in different organ systems are summarized in Table 1. A schematic overview of these actions across organ systems is provided in Figure 3.

### 7.1. Cardiovascular Function

CGRP constitutes one of the most potent endogenous vasodilators, with a potency approximately ten times greater than that of prostaglandins and 10 to 1000 times greater than that of other vasoactive peptides. CGRP-mediated vasodilation results from two complementary mechanisms that include direct activation of receptors on vascular smooth muscle cells and indirect stimulation via nitric oxide release from endothelial cells. Studies in humans using intravenous infusion of human CGRP-α have shown that the peptide produces dilation of both cerebral and extracranial arteries, with maximal vascular changes observed between 15 and 20 min after the start of infusion and persisting between 20 and 120 min depending on the dose administered. Infusion of CGRP in healthy volunteers induces characteristic hemodynamic effects including facial flushing, feeling of warmth, palpitations, and increased heart rate, accompanied by a reduction in systemic vascular resistance and consequently in blood pressure. This role of CGRP is supported by its widespread sensory innervation of the vasculature, where the peptide is released in response to stimuli such as mechanical distension, hypoxia, and inflammatory mediators, exerting compensatory vasodilatory effects that contribute to homeostatic cardiovascular regulation [1,9,52].

### 7.2. Nociception and Pain Transmission

CGRP plays an important role in modulating nociception and pain processing both peripherally and centrally, being especially relevant in the development and maintenance of neuronal sensitization states.

At the peripheral level, CGRP sensitizes primary nociceptors by reducing the activation threshold of TRPV1 and other TRP family channels through PKA-dependent phosphorylation, increasing neuronal responsiveness to thermal and mechanical stimuli [1,8]. At the spinal level, CGRP released from central terminals of primary afferents facilitates glutamatergic transmission to second-order neurons through both presynaptic enhancement of glutamate release and postsynaptic potentiation of excitatory responses, thereby establishing states of central sensitization characterized by hyperalgesia and allodynia that underlie chronic pain conditions [5,8].

### 7.3. Neurogenic Inflammation and Immune Function

CGRP constitutes a mediator of neurogenic inflammation, a process by which activation of peripheral sensory neurons produces antidromic release of neuropeptides that induce local inflammatory responses. CGRP-mediated neurogenic inflammation is characterized by arteriolar vasodilation, increased microvascular permeability, and extravasation of plasma proteins, phenomena that give rise to tissue edema formation [53]. CGRP exerts complex effects on immune cells, including mast cells, macrophages, neutrophils, and lymphocytes, and these effects depend heavily on the tissue context and activation state of the target cells [54,55,56]. Mast cells, which are frequently located close to CGRP-containing sensory nerve terminals, represent important effector cells in neurogenic inflammation [57]. Activation of mast cells by CGRP produces the release of proinflammatory mediators, including histamine, cytokines such as TNF-α, GM-CSF, and IL-8, and chemokines that recruit additional immune cells to the site of neuronal activation [56,57].

The molecular mechanism by which CGRP activates mast cells involves not only the classical CLR-RAMP1 receptor but also activation of Mrgprb2 in mice and its human homolog MRGPRX2, although the latter respond mainly to substance P rather than CGRP [58,59,60]. The functional interaction between sensory neurons and mast cells mediated by neuropeptides establishes a feedback circuit that amplifies inflammatory and painful responses. Macrophages represent another immune population modulated by CGRP, although the effects of the peptide on these cells can be either proinflammatory or antiinflammatory depending on context. In the context of infections, CGRP can promote antiinflammatory responses by inducing antiinflammatory cytokines and M2 polarization markers in macrophages, suggesting a protective role of the peptide in inflammation resolution. In the cranial dura mater, CGRP released from perivascular trigeminal nerve terminals interacts with dural macrophages and mast cells to generate sterile meningeal inflammation characterized by mast cell degranulation, local vasodilation, and increased microvascular permeability, which sensitizes perivascular trigeminal nociceptors through a mechanism mechanistically distinct from systemic neurogenic inflammation and directly relevant to migraine pathophysiology [61,62,63].

### 7.4. Gastrointestinal Function

CGRP exerts multiple functions in the gastrointestinal tract, where it participates in the regulation of motility, secretion, blood flow, and visceral nociception. Sensory innervation of the gastrointestinal tract by CGRP-containing neurons is abundant, with CGRP-positive nerve fibers present in all layers of the intestinal wall, including the mucosa, submucosa, and muscle layers. CGRP modulates gastrointestinal motility through effects on enteric nervous system neurons and through direct actions on smooth muscle cells, these effects being biphasic and region-dependent along the gastrointestinal tract. In the small intestine, CGRP induces phasic contractions of circular muscle through stimulation of cholinergic neurons, whereas in the colon it can exert both excitatory and inhibitory effects depending on whether it acts on longitudinal or circular muscle. Analysis of the stimulatory effect of CGRP on intestinal propulsion has revealed that intrinsic primary afferent neurons expressing CGRP play an important role in initiating enteric reflexes that include ascending contraction and descending relaxation of circular muscle, movements essential for peristalsis [64,65,66].

Mechanistic studies indicate that constipation induced by blockade of the CGRP system results from interference with the physiological function of the peptide in the small and large intestine, where it contributes to maintaining peristaltic motor activity, secretion of ions and water into the intestinal lumen, and intestinal transit. CGRP stimulates secretion of ions and water in the intestine through actions on epithelial cells and on secretomotor neurons, and the motor-stimulating and prosecretory actions of CGRP combine to accelerate intestinal transit, an activity profile that has been confirmed by the ability of CGRP to induce diarrhea in mice, dogs, and humans when administered at high doses. The protective function of CGRP in the gastrointestinal mucosa has been demonstrated in animal models of intestinal ischemic injury, where the peptide participates in the modulation of intestinal blood flow, sensorimotor activity, and tissue oxygenation [64,65,66].

### 7.5. Bone Metabolism and Remodeling

CGRP plays regulatory roles in bone metabolism by modulating the balance between osteoblast and osteoclast activity, cells responsible for bone formation and resorption, respectively. Sensory innervation of bone tissue by CGRP-containing nerve fibers is abundant, with CGRP-positive nerve terminals present in the periosteum, cortical bone, and bone marrow, establishing close contacts with osteoprogenitor cells and mature bone cells. Studies in transgenic mice overexpressing CGRP in osteoblasts have shown that increasing local CGRP levels produces an increase in bone density, indicating an anabolic effect of the peptide on bone. The mechanism by which CGRP stimulates bone formation involves promotion of osteoblastic proliferation and differentiation, as well as increased expression of osteogenic markers such as osteocalcin, alkaline phosphatase, and Runx2. CGRP activates osteocalcin transcription through phosphorylation of transcription factors of the ATF4 family, which are regulators of terminal osteoblastic differentiation [67,68,69].

In parallel with its effects on osteoblasts, CGRP inhibits osteoclastogenesis and bone resorption by modulating the RANKL-OPG system, which constitutes the main regulator of osteoclast differentiation and activation. CGRP reduces RANKL expression in osteoblastic cells while simultaneously increasing expression of osteoprotegerin, a decoy protein that neutralizes RANKL and prevents its binding to RANK on osteoclast precursors. This shift in the RANKL/OPG balance toward an anti-resorptive profile results in inhibition of monocyte differentiation into mature osteoclasts and reduction in the resorptive activity of pre-existing osteoclasts. The effects of CGRP on bone metabolism vary with age, being especially relevant in aging, where a decrease in CGRP levels in the bone marrow microenvironment is observed. Studies have shown that reduced CGRP levels in aged mice correlate with a shift in the differentiation fate of bone marrow mesenchymal stem cells from the osteoblastic lineage toward the adipocytic lineage, a process that contributes to age-related osteoporosis. Exogenous administration of CGRP to aged mice and ovariectomized mice accelerates bone formation in vivo, suggesting that the CGRP system could represent a therapeutic target for osteoporosis prevention [67,68,69]. CGRP also promotes bone defect regeneration by inducing ANGPTL4 secretion from bone blood vessels, which in turn drives osteogenic differentiation of bone marrow mesenchymal stem cells [70]. Furthermore, local IL-6 blockade at fracture sites enhances CGRP expression and accelerates bone healing, indicating a reciprocal regulation between IL-6 and CGRP signaling [71].

### 7.6. Wound Healing and Tissue Regeneration

CGRP exerts effects on tissue repair and regeneration processes through modulation of immune responses, promotion of angiogenesis, and regulation of cell migration and function [72,73,74]. Recent studies have shown that ablation of Nav1.8-expressing nociceptors, which release CGRP, impairs cutaneous wound healing and muscle regeneration after acute injuries [69,72,73]. The mechanism by which CGRP promotes tissue healing involves inhibitory effects on excessive migration and activation of myeloid cells, particularly neutrophils and monocytes-macrophages, which when persistently over-present in injured tissues can interfere with proper repair. CGRP markedly inhibits neutrophil and macrophage migration toward common chemokines present in wounds, such as CXCL1 for neutrophils and CCL2 for macrophages, thereby reducing excessive recruitment of these cells to injury sites. Furthermore, CGRP promotes apoptosis of neutrophils and macrophages in injured tissues, facilitating early clearance of proinflammatory cells and accelerating the transition to the repair phase [69,72,73,75].

Exogenous administration of CGRP, particularly of variants with greater stability, improves both cutaneous wound closure and muscle regeneration, reducing the number of neutrophils and monocytes-macrophages in the early stages after injury and modulating macrophage phenotype toward an antiinflammatory profile characterized by reduced levels of the proinflammatory marker Ly6C and increased levels of the antiinflammatory marker CD206 [5,17,76]. In diabetic wound models, sustained-release CGRP microspheres have been shown to accelerate healing by promoting M2 macrophage polarization and enhancing neurovascular regeneration [77].

The effect of CGRP on angiogenesis, an essential process for tissue repair, has been documented in multiple experimental contexts, including periodontal regeneration [1,5,17,78]. Studies have shown that CGRP can promote angiogenesis through mechanisms involving both direct effects on endothelial cells and indirect effects through modulation of the tissue microenvironment [1,5,17,78]. In the context of periodontal regeneration, CGRP promotes the formation of vascular networks that establish the blood supply necessary for the regeneration of periodontal tissues in tissue defects [1,5,17,78]. The effects of CGRP on soft tissue repair are consistent with previous observations documenting the ability of the peptide to facilitate healing in multiple experimental models [1,5,17,78].

### 7.7. Respiratory Function

CGRP exerts multiple effects on the respiratory system, where it modulates airway tone, bronchial reactivity, and pulmonary inflammatory responses [79,80]. Sensory innervation of the bronchial tree by CGRP-containing fibers is abundant, with nerve terminals present in the epithelium, bronchial smooth muscle, and in association with pulmonary blood vessels. The effects of CGRP on bronchial smooth muscle tone are complex and context-dependent, with evidence of both bronchodilator and bronchoconstrictor actions under different experimental conditions. In studies with isolated human bronchi, CGRP-α induces bronchodilation through activation of receptors on smooth muscle cells, an effect that is blocked by the antagonist CGRP 8–37. This bronchodilator effect of CGRP is presumed to be mediated by PKA activation and subsequent smooth muscle relaxation, a mechanism analogous to that mediating vasodilation in blood vessels [1,80].

The role of CGRP in asthma pathophysiology has received attention, with evidence suggesting that the peptide may contribute to both bronchoconstrictor responses and inflammatory processes in this condition [79,80,81]. CGRP levels are elevated in patients with asthma, and the main source of CGRP in the asthmatic lung appears to be pulmonary neuroendocrine cells, which are also increased in these patients [79,81]. CGRP can increase mast cell activity, promote eosinophil recruitment through induction of IL-5, and facilitate recruitment and responses of Th2 cells through induction of CCL17 and CCL22, while simultaneously inhibiting CXCL9 and CCL10, suggesting a proinflammatory role of the peptide in asthma [79,82]. Studies in RAMP1-deficient mice have confirmed the contribution of CGRP to asthmatic inflammatory responses, and treatment with the CGRP receptor antagonist rimegepant in a murine model of asthma produced a decrease in airway inflammation and goblet cell number [79,80]. Hyperpnea-induced bronchoconstriction in guinea pigs, which constitutes a model of exercise-induced asthma in humans, appears to involve release of CGRP from capsaicin-sensitive sensory nerves and its interaction with mast cells and bronchial smooth muscle.

### 7.8. Thermoregulation

CGRP participates in the regulation of body temperature through effects on thermosensitive neurons in the preoptic area of the hypothalamus, the nucleus for thermoregulatory control [83,84]. Administration of CGRP, either systemically or directly into the dorsomedial or ventromedial hypothalamus, induces hyperthermia characterized by increases in oxygen consumption, heart rate, and core temperature in rats [85]. The mechanism by which CGRP generates this hyperthermic shift in thermoregulatory control involves selective effects on the firing rates of thermosensitive and thermoinsensitive neurons in the preoptic area [84]. Electrophysiological studies have shown that CGRP decreases the firing rates of all heat-sensitive neurons examined, while increasing the firing rates of most thermoinsensitive neurons [84]. These differential effects on thermoregulatory neuron populations are consistent with a model in which the decrease in inhibitory activity from heat-sensitive neurons, combined with the increase in activity of thermoinsensitive neurons projecting to effector regions, would result in activation of heat-production mechanisms and inhibition of heat-loss mechanisms [84,86].

CGRP-induced hyperthermia differs from fever in several aspects, being generally of shorter duration and resulting from both increases in sympathetic activation and metabolic rate [86]. This CGRP-mediated hyperthermic response represents a frequent complication of antiandrogen treatment for prostate cancer in men, a condition that correlates with elevations in CGRP levels [87,88,89]. Experimental injection of CGRP in humans and animals produces similar increases in sympathetic activation and skin temperature, confirming the physiological relevance of the thermoregulatory effects of the peptide [90,91,92]. The mechanism by which CGRP can stimulate a hyperthermic shift in body temperature appears analogous to the mechanism by which prostaglandin E2 mediates the fever accompanying immune system stimulation, albeit with distinct temporal kinetics [84,86].

**Table 1:** Summary of physiological functions of CGRP by system.

System/Tissue	Main Function(s)	Key Mechanisms of Action	Pathophysiological Relevance
Cardiovascular [9,93,94]	Potent vasodilation, cardioprotection	Activation of receptors on smooth muscle and endothelium; K(ATP) channel opening; NO production.	Blood pressure regulation; protection against ischemia; hypotension as an adverse effect of blockers.
Nociceptive [12,43,95]	Nociceptor sensitization, facilitation of pain transmission	Sensitization of TRPV1 channels; facilitation of glutamate release in the dorsal horn.	Central role in migraine pathophysiology and chronic pain.
Gastrointestinal [10,64,96]	Regulation of motility and secretion	Stimulation of enteric neurons and smooth muscle cells; pro-secretory action on epithelium.	Constipation is a common adverse effect of CGRP antagonists.
Bone [69,70,71]	Bone anabolism (stimulates osteoblasts), inhibition of resorption (inhibits osteoclasts)	Activation of Runx2 and ATF4; modulation of the RANKL/OPG balance.	Potential target for treating osteoporosis.
Tissue Regeneration [17,70,77]	Promotes wound healing and angiogenesis	Inhibition of excessive myeloid cell migration; promotion of neutrophil apoptosis.	Potential therapeutic application in chronic wounds.
Metabolism [12,97,98]	Modulation of energy expenditure and glucose homeostasis	Inhibition of sympathetic activity (contributing to obesity in animal models).	CGRP blockade could improve insulin resistance in the context of obesity.

### 7.9. Energy Metabolism and Glucose Homeostasis

CGRP exerts modulatory effects on energy metabolism and glucose homeostasis, these effects being especially evident under conditions of obesity and metabolic challenge [85,98,99]. Studies in CGRP-deficient mice have revealed that the absence of the endogenous peptide confers resistance to high-fat diet-induced weight gain, suggesting that endogenous CGRP exerts unfavorable metabolic effects in the context of obesity [98,100]. CGRP-deficient mice fed a high-fat diet show better glucose tolerance and greater insulin sensitivity compared to control mice fed the same diet, although these differences are not evident when both groups consume a normal diet [98]. Analysis of expired gases revealed higher oxygen consumption in CGRP-deficient mice, indicating increased energy expenditure that could contribute to their resistance to weight gain [98,100]. At the adipose tissue level, CGRP-deficient mice exhibit suppression of adipocyte hypertrophy together with increased expression of β-3-adrenergic receptor, hormone-sensitive lipase, and adiponectin, consistent with increased lipolytic activity [98].

Loss of the inhibitory effect of CGRP on sympathetic nervous system activity could contribute to the metabolic changes observed in CGRP-deficient mice, resulting in increased lipolysis and elevated energy expenditure [98]. CGRP-deficient mice present reduced fasting hyperglycemia, attenuated hyperinsulinemia, and significantly lower serum leptin levels compared to control mice fed a high-fat diet, although serum concentrations of triglycerides, free fatty acids, and total cholesterol remain similar between the two groups [98]. Additional correlative evidence demonstrates that loss of CGRP-containing sensory fibers is associated with increased insulin secretion and improved glucose tolerance in certain experimental contexts [85]. The effects of monoclonal antibodies against CGRP on body weight and metabolic parameters have been evaluated in murine models of obesity, demonstrating that treatment with these antibodies significantly reduces high-fat diet-dependent weight gain and adiposity [97,99]. The peripheral mechanism by which anti-CGRP antibodies reduce glucose levels and weight gain could involve combined modulation of glucose-stimulated insulin secretion, as well as reduction in adiposity [97].

## 8. Sensory System, Nociception, and the Trigeminovascular System

The trigeminovascular system constitutes an anatomical and functional structure comprising sensory innervation of the cranial meninges, large intracranial vessels, and dural venous sinuses by nociceptive fibers originating in the trigeminal ganglion and the upper cervical ganglia [43]. The sensory neurons that make up this system are predominantly of small and medium diameter, corresponding to unmyelinated C fibers and thinly myelinated Aδ fibers, which abundantly express CGRP together with other neuropeptides such as substance P [8]. The distribution of CGRP-positive nerve terminals in the meninges is densest in the dura mater, where these fibers form perivascular plexuses around the meningeal arteries, establishing an anatomical localization suitable for detecting changes in the vascular environment and for modulating vascular tone through neuropeptide release [43]. The central projections of these sensory neurons terminate in the trigeminal nucleus caudalis and in the dorsal horns of the upper cervical segments C1 and C2, forming the trigeminocervical complex that integrates nociceptive information from the cranial territory [8].

The importance of the trigeminovascular system in migraine pathophysiology has been established through multiple lines of evidence including clinical observations, experimental provocation studies, and the therapeutic efficacy of interventions targeting the CGRP system [6]. Studies measuring CGRP levels in the external jugular circulation during spontaneous migraine attacks revealed increases in the peptide on the side ipsilateral to the pain, elevations that normalized after successful treatment with sumatriptan. Intravenous infusion of CGRP in patients with migraine induces attacks with characteristics identical to spontaneous attacks in approximately 60 percent of patients, whereas in healthy control subjects the infusion produces only mild transient headache without migraine features [6,101,102]. This provocative effect of exogenous CGRP occurs with a delay of several hours after infusion, suggesting that the peptide triggers complex pathophysiological cascades rather than directly inducing pain through acute activation of nociceptors [5,6]. Despite the success of CGRP-targeted therapies, a significant critical challenge remains: approximately 40–50% of patients do not achieve a 50% reduction in migraine days, suggesting that CGRP is not the sole driver for all migraine phenotypes [12,103]. Furthermore, the ‘wearing-off’ effect—an increase in headache frequency toward the end of a monthly dose cycle—remains an unresolved clinical concern requiring further optimization of dosing regimens [5,6,18].

**Figure 4:**
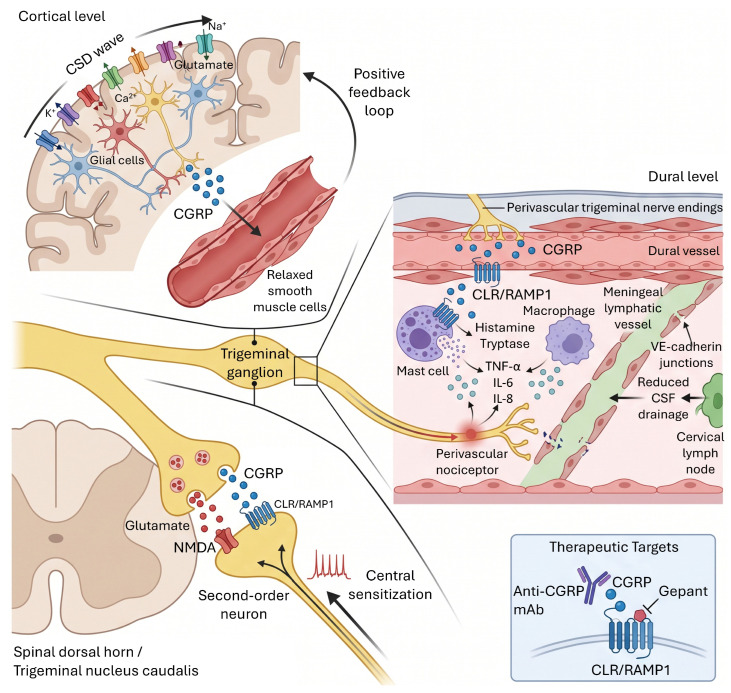
Contribution of CGRP to migraine pathophysiology at the trigeminovascular level. Migraine is a complex neurological disorder in which CGRP acts as a central mediator. Intravenous CGRP infusion triggers migraine-like attacks in approximately 60% of patients, and peptide levels are elevated in ipsilateral external jugular blood during spontaneous attacks. Current pathophysiological models recognize several levels of action: (1) Cortical spreading depression (CSD)—a wave of neuronal depolarization that propagates across the cortex—triggers parenchymal CGRP release, which in turn dilates parenchymal arterioles and lowers the threshold for subsequent CSD waves, establishing a positive feedback loop. (2) In the dura mater, CGRP released from perivascular trigeminal nerve endings acts on dural mast cells and macrophages, inducing degranulation and release of proinflammatory cytokines (TNF-α, IL-6, IL-8), generating sterile neurogenic inflammation that sensitizes perivascular nociceptors. (3) CGRP also regulates meningeal lymphatic vessel permeability by remodeling VE-cadherin junctions, reducing cerebrospinal fluid drainage to cervical lymph nodes—a phenomenon associated with pain chronification. (4) In the spinal dorsal horn and trigeminal nucleus caudalis, CGRP facilitates glutamate release and potentiates second-order neuron responses, contributing to central sensitization. The therapeutic efficacy of anti-CGRP monoclonal antibodies and gepants confirms the relevance of this neuropeptide as a target for acute and preventive migraine treatment.

The traditional trigeminovascular hypothesis proposes that activation of meningeal nociceptors triggers the release of vasoactive neuropeptides, particularly CGRP, which induce intracranial vasodilation and neurogenic inflammation, thereby contributing to migraine pain [101]. However, a more recent hypothesis, termed the vaso-neuronal trigeminovascular hypothesis, proposes that intracranial vasodilation may act as a primary initiator of migraine attacks through mechanical and chemical activation of perivascular nociceptors. According to this model, vasodilation of the meningeal arteries, particularly the middle meningeal artery, generates mechanical stimuli that activate perivascular trigeminal nerve terminals, which in turn release signaling molecules such as CGRP that mediate migraine pathogenesis. Evidence in favor of this hypothesis includes the observation that during CGRP-induced migraine attacks, both the middle meningeal artery and the middle cerebral artery dilate ipsilaterally in patients with unilateral headache, and bilaterally in those with bilateral headache [102]. Intravenously administered CGRP does not directly dilate the middle cerebral artery, implying that the dilation observed during the migraine attack reflects processes linked to the attack itself rather than direct pharmacological effects of circulating CGRP [102] (Figure 4).

Characterization of the functional properties of dural nociceptors has revealed that these neurons exhibit mechanosensitivity, responding to mechanical deformation of the dura mater with action potential discharges whose frequency is proportional to the magnitude of the stimulus [104]. Electrophysiological experiments performed by extracellular recording of dural afferent fibers in animal models have shown that approximately 85 percent of these neurons respond to mechanical stimulation applied to the dura mater, with activation thresholds ranging between 0.5 and 2 g of force [95]. Local application of proinflammatory mediators such as prostaglandin E2, bradykinin, serotonin, and histamine sensitizes these meningeal nociceptors, reducing their mechanical response threshold and increasing their spontaneous firing rate [95]. These phenomena of peripheral sensitization could contribute to the cephalic cutaneous allodynia observed during migraine attacks, where normally innocuous stimuli such as hair brushing are perceived as painful [8]. Contrary to expectation, application of CGRP directly onto the dura mater does not produce significant effects on spontaneous activity or on mechanically evoked responses in dural nociceptors, suggesting that the effects of CGRP in migraine may be more complex than simple direct activation of meningeal nociceptors [95].

Recent studies have identified a previously unrecognized role of meningeal lymphatic vessels in the pathophysiology of CGRP-mediated chronic migraine [5]. CGRP signaling in meningeal lymphatic vessels regulates endothelial permeability through rearrangement of membrane junction proteins, particularly VE-cadherin, resulting in reduced flow of cerebrospinal fluid to the cervical lymph nodes [5]. In a murine model of chronic migraine induced by repeated administration of nitroglycerin, increased CGRP levels were observed adjacent to meningeal lymphatic vessels, accompanied by the formation of continuous VE-cadherin junctions at the capillary terminal points of these vessels [5]. These structural changes were associated with reduced clearance of cerebrospinal fluid and with alterations in interactions between meningeal lymphatic vessels and immune cells. Selective blockade of CGRP signaling in meningeal lymphatic vessels attenuated pain-associated behavior in acute migraine in rodents, suggesting that meningeal lymphatic vessels represent an effector target of CGRP within the trigeminovascular system [5]. This observation could help explain why only approximately 50 percent of patients treated with the highest dose of erenumab experience a greater than 50 percent reduction in monthly migraine days, correlating with the partial attenuation of migraine symptoms observed in mice with impaired lymphatic response to CGRP [5].

CGRP plays important roles in the development and maintenance of peripheral sensitization, a state of hyperresponsiveness of nociceptors to normal and subthreshold stimuli that contributes to persistent pain [8]. Activation of peripheral free nerve endings by noxious stimuli or by inflammatory mediators generates axon reflexes that cause release of CGRP and substance P from adjacent terminals of the same neuron, leading to evoked release of additional proinflammatory mediators that amplify nociceptive responses. Locally released CGRP directly sensitizes nociceptors by modulating the expression and function of multiple ion channel types that determine neuronal excitability [8]. Electrophysiological studies in dorsal root ganglion neurons have demonstrated that application of CGRP reduces the activation threshold of TRPV1 channels, increasing the response to noxious thermal stimuli and to capsaicin. This sensitizing effect of CGRP on TRPV1 is mediated by PKA activation, which phosphorylates specific serine residues on the channel, modifying its voltage and ligand sensitivity [8]. The role of TRPV1 in regulating CGRP expression has been confirmed by studies in *Trpv1*-deficient mice, which do not exhibit thermal hyperalgesia associated with Freund’s complete adjuvant-induced inflammation and show marked attenuation of CGRP upregulation induced by acidic conditions [14].

Activation of TRPV1 by protons in acidic inflammatory environments triggers an intracellular signaling cascade that culminates in increased CGRP expression in sensory neurons [1,8,14]. The molecular mechanism involves calcium entry through activated TRPV1, which activates calcium-calmodulin-dependent kinase, which in turn phosphorylates the transcription factor CREB at serine 133 [8,13,14,105]. Phosphorylated CREB binds to cAMP response elements in the promoter region of the CGRP gene, increasing transcription of the peptide [5,8,105]. This TRPV1-CaMK-CREB-CGRP signaling pathway represents a positive feedback mechanism by which activation of nociceptors in inflammatory environments increases CGRP expression, which in turn further sensitizes nociceptors to subsequent noxious stimuli [8,14,43,105]. Experiments with specific pharmacological inhibitors have confirmed that both CaMK activation and CREB phosphorylation are necessary steps in the TRPV1 activation-induced upregulation of CGRP [8,105].

Release of CGRP from central terminals of primary sensory neurons in the dorsal horn of the spinal cord and the trigeminal nucleus caudalis plays an important role in the establishment and maintenance of central sensitization [8]. Central sensitization is characterized by an increase in the excitability of second-order neurons in the central nociceptive system, such that input stimuli that would normally be subthreshold are sufficient to generate action potentials and painful responses. CGRP released in the dorsal horn facilitates nociceptive synaptic transmission through multiple mechanisms including presynaptic effects that increase glutamate release from primary afferent terminals, as well as postsynaptic effects that potentiate the response of second-order neurons to excitatory neurotransmitters [95]. Studies in brainstem slice preparations have demonstrated that basal release of CGRP increases the excitability of trigeminal sensory neurons through activation of CGRP1 receptors, an effect that is blocked by the antagonist CGRP 8–37. This increase in neuronal excitability mediated by CGRP may contribute to the development of chronic pain states in which nociceptive transmission is pathologically facilitated [95].

Cortical spreading depression constitutes a key pathophysiological phenomenon in migraine with aura and represents a potential triggering mechanism for activation of the trigeminovascular system [5]. Originally described by Leão in 1944, consists of a wave of electrical activity characterized by excitation followed by depression that propagates across the cerebral cortex after electrical or chemical stimulation [5]. The central event in the generation and propagation of cortical spreading depression is a marked decrease in neuronal membrane resistance associated with massive increases in extracellular potassium, sustained neuronal depolarization, and hyperemia followed by oligohemia [5]. Experiments in neocortical slices revealed that the CGRP receptor antagonist olcegepant increases the threshold for induction of cortical spreading depression by electrical stimulation, suggesting that blockade of CGRP signaling stabilizes neuronal membranes against the massive depolarization that characterizes this phenomenon [5].

The relationship between CGRP and cortical spreading depression is bidirectional. Thus, cortical spreading depression triggers parenchymal CGRP release, which in turn dilates parenchymal arterioles and lowers the threshold for subsequent depolarization waves, establishing a positive feedback loop that sustains neuronal activation and contributes to migraine pathophysiology [5]. Cortical spreading depression can also activate the trigeminal nociceptive system both peripherally, through release of mediators from the brain parenchyma acting on meningeal nociceptors, and centrally, through activation of neurons in the trigeminal nucleus caudalis. The ability of CGRP to act as a physiological vasodilatory agent within the central nervous system in vivo has been recently demonstrated, providing direct evidence that CGRP can influence cerebral vascular tone [5].

Vestibular migraine is a subtype characterized by recurrent vertigo and migrainous symptoms. CGRP is expressed in central and peripheral vestibular structures, and its infusion in rodents causes motion sickness and imbalance. Emerging clinical evidence suggests that CGRP-targeted therapies, including gepants, may be effective in reducing vertigo and dizziness in these patients, although controlled trials are still needed [106,107]. Similarly, given the shared central sensitization pathways with migraine, CGRP signaling has been implicated in the pathophysiology of tinnitus, suggesting that CGRP-targeted medications could modulate the intensity of perceived sound [108].

## 9. Pathophysiology

Beyond its well-established role in migraine, CGRP contributes to the pathophysiology of various other diseases, including chronic pain, osteoarthritis, cancer, cardiovascular disorders, sepsis, and neurodegeneration. The emerging pathophysiological roles of the CGRP system are summarized in Table 2. A summary of CGRP-related pathologies categorized by the strength of clinical and preclinical evidence is shown in Table 3.

### 9.1. Chronic Pain and Neuropathic Conditions

CGRP plays a pathophysiological role in multiple chronic pain syndromes including neuropathic pain, inflammatory pain, visceral pain, and musculoskeletal pain [1,8,13]. Clinical studies have documented elevations in CGRP levels in plasma, synovial fluid, and cerebrospinal fluid in patients with musculoskeletal pain conditions, with positive correlations observed between peptide concentrations and pain intensity [95]. In patients with chronic knee pain secondary to osteoarthritis, elevated CGRP levels have been detected both in serum and in synovial fluid compared to controls, with a positive correlation between serum CGRP levels and pain intensity assessed by visual analog scales [95]. Patients with chronic low back pain due to osteoarthritis show decreased blood CGRP levels compared to healthy controls, although this apparent discrepancy could reflect depletion of circulating CGRP due to its release and utilization at sites of local inflammation. Biopsies of intervertebral disks from patients with degenerative disk disease revealed increases in CGRP compared to post-mortem control disks, and intervertebral disc biopsies from patients with low back pain contained CGRP-immunoreactive nerve fibers [95].

Complex regional pain syndrome represents a chronic pain condition characterized by disproportionate pain, neurogenic inflammation, and vasomotor and trophic changes typically affecting a limb after traumatic injury [1]. Clinical studies in patients with acute complex regional pain syndrome have demonstrated elevated systemic CGRP levels compared to healthy controls and to patients with other limb injuries not developing the syndrome, suggesting that CGRP-mediated neurogenic inflammation contributes as a pathophysiological mechanism to the vasodilation, edema, and increased sweating characteristic of this condition [1]. Experiments in animal models have confirmed that neuropeptides, particularly substance P and CGRP, mediate the increased neurogenic inflammation and pain in complex regional pain syndrome [8]. Dysfunction of neuropeptide-containing primary C afferent fibers leads to the vascular symptoms, trophic changes, and generation of pain in this condition. During the neuroinflammation associated with complex regional pain syndrome, generated neuropeptides and neurotransmitters can activate microglia and astrocytes, leading to a cascade of glial mediators that sensitize neurons and impact synaptic plasticity, establishing a cyclic dialog between microglia, astrocytes, and neurons that sustains central nociceptive sensitization and neuroinflammation [8].

Chemotherapy-induced neuropathic pain constitutes a dose-limiting complication in oncological treatment, affecting 30 to 40 percent of patients treated with agents such as paclitaxel, oxaliplatin, and vincristine [12,17,109]. The pathophysiological mechanisms of chemotherapy-induced neuropathic pain are multifactorial and include direct damage to peripheral nerves, mitochondrial dysfunction in sensory neurons, increased reactive oxygen species, activation of inflammatory cascades, and both peripheral and central sensitization [13,14,17,109]. The CGRP system contributes to the development and maintenance of chemotherapy-induced neuropathic pain through multiple mechanisms including facilitation of nociceptive synaptic transmission in the dorsal horn of the spinal cord and sensitization of peripheral nociceptors [5,8,17,110]. Studies in murine models of paclitaxel-induced peripheral neuropathy have demonstrated upregulation of CGRP receptor components, specifically CLR and RAMP1, in the ipsilateral spinal dorsal horn after drug administration, with these increases correlating temporally with the development of mechanical allodynia [5,17,110]. However, it has been found that in a chronic oxaliplatin-induced neuropathy model, CGRP expression in dorsal root ganglia was not increased but rather decreased, suggesting that enhanced CGRP signaling may result from increased calcium-dependent release and receptor sensitization rather than upregulation [111].

### 9.2. Osteoarthritis and Joint Diseases

CGRP exerts dual and seemingly contradictory functions in the pathophysiology of osteoarthritis, contributing to proinflammatory and catabolic processes in articular cartilage while exerting protective effects on subchondral bone structures [5]. Studies in murine models of age-dependent osteoarthritis have revealed that absence of CGRP-α prevents cartilage degradation in the knee joint and decreases expression of proinflammatory and catabolic cartilage markers. However, aged CGRP-α-deficient mice exhibited signs of subchondral tibial bone sclerosis, deteriorated bone quality in the epiphysis and metaphysis, and marked loss of trabecular bone, indicating that CGRP exerts a bone-protective role in primary osteoarthritis [5]. This dual, proinflammatory and bone-protective role of CGRP in primary osteoarthritis is similar to the effects observed in experimental rheumatoid arthritis [1,5]. Intra-articular CGRP can be secreted by fibroblast-like synoviocytes and its expression correlates with pain in osteoarthritis. The effects of CGRP on cartilage appear to depend on the prior phenotype of chondrocytes, with a chondroprotective and anti-apoptotic response observed when CGRP is added to healthy chondrocytes, whereas when added to osteoarthritis-derived chondrocytes, markers of collagen formation and glycosaminoglycans are markedly reduced [5].

Increased mechanosensitivity in osteoarthritic knees and pain behavior can be reduced by peripherally acting CGRP receptor antagonists [5,8]. The effects of CGRP pathway blockade on arthritic joint afferents, but not on normal joints, suggest contributions to sensitization rather than to normal joint nociception, indicating that CGRP could contribute to the transition from normal synovitis to persistent synovitis and from nociception to sensitization [5,8,112]. Angiogenesis is a feature of chronic inflammation and, in both osteoarthritis and rheumatoid arthritis, affects multiple joint tissues, including the synovium, osteochondral junction, and meniscus [5,8,112]. In the synovium, angiogenesis occurs concurrently with vascular regression, resulting in vascular redistribution away from the synovial lining, and new blood vessels also grow into uncalcified articular cartilage and the inner two-thirds of the knee meniscus, structures that are avascular in the normal joint. Clinical trials with the anti-CGRP monoclonal antibody galcanezumab in patients with osteoarthritis knee pain did not demonstrate pain improvement compared to placebo, suggesting that systemic CGRP blockade may not be sufficient to control osteoarthritis pain or that pain mechanisms in this condition are more complex than simple CGRP signaling [5,8,12,113].

### 9.3. Cancer and Bone Metastases

Endogenous CGRP facilitates tumor-associated angiogenesis and tumor growth through mechanisms involving release of the peptide from neuronal systems, including primary sensory neurons [1,5,12,16,114]. Studies in CGRP-deficient mice implanted with Lewis lung carcinoma cells revealed that tumor growth and tumor-associated angiogenesis are reduced compared to wild-type mice [5,16,114]. CGRP increases tube formation by endothelial cells in vitro and enhances sponge-induced angiogenesis in vivo, showing direct proangiogenic capacity [1,5,16,114]. Treatment with the CGRP receptor antagonist 8–37 can block tumor-associated angiogenesis and tumor growth, indicating that development of CGRP receptor antagonists could be an anticancer therapeutic strategy [1,5,16,114]. The phenotype observed in tumors during blockade of CGRP or its release was reduced formation of stromal tissues and, given that stromal cells could derive from bone marrow, it is possible that CGRP regulates some signaling relevant to the recruitment of these cells, potentially becoming a target for controlling tumor-associated angiogenesis.

CGRP-expressing sensory nerves induce progression of bone metastases through CLR expressed in bone-metastatic cancer cells, activating the p38/HSP27 pathway [12,16,115]. In oral squamous cell carcinoma, CGRP signaling from sensory neurons promotes tumor growth and immune evasion by limiting anti-tumor immunity, and systemic CGRP receptor antagonism slows tumor progression [116]. Clinical studies have demonstrated that cancer patients with bone metastatic disease have elevated serum CGRP levels compared to those without bone metastases. Bone metastatic tumor samples express higher levels of CLR compared to samples from other metastases, primary tumors, or benign tissues, and CLR expression in tumors correlates negatively with recurrence-free survival of cancer patients [12,16,115]. Experiments in murine models demonstrated that CGRP-expressing sensory nerves are enriched in the periosteum of mice with bone metastases, and that blockade of sensory nerve-derived CGRP by anti-CGRP monoclonal antibody can reduce bone metastasis progression in vivo. CGRP induces proliferation of cancer cells through the CLR/p38/HSP27 pathway, providing a molecular mechanism explaining how CGRP-rich sensory innervation of the bone microenvironment can promote metastatic tumor growth [12,16,115]. These data suggest that CGRP-expressing sensory nerves are involved in bone metastatic progression and that the CGRP/CLR axis may serve as a potential therapeutic target for controlling bone metastases.

### 9.4. Cardiovascular Diseases

The antihypertensive role of endogenous CGRP is evidenced by the spontaneous hypertension and exaggerated hypertensive response to angiotensin II observed in CGRP-α-deficient mice, and by the upregulation of RAMP1 and CGRP expression in murine models of hypertension and heart failure, indicating a compensatory cardioprotective activation of the system under pathological conditions [1,5,9,94]. In patients with hypertension, dysfunctional nitric oxide production and endothelial dysfunction can be partially compensated by CGRP, which promotes restoration of blood flow in mesenteric resistance vessels and protects against cardiovascular remodeling through the canonical CLR-RAMP1 receptor [5,9].

CGRP possesses positive chronotropic and inotropic activity, in addition to its ability to cause vasodilation and consequently hypotension, and the peptide has been described to increase contractile force through stimulation of the sympathetic nervous system [1,5]. Patients with congestive heart failure showed improvement in cardiac contractility after receiving CGRP-β infusion for 24 h [5]. The cardioprotective effects of CGRP also include protection against myocardial ischemia–reperfusion injury, modulation of pathological cardiac remodeling, and regulation of endothelial function, through mechanisms that include activation of ATP-sensitive potassium channels, induction of heat shock proteins, and modulation of apoptotic pathways [1,5,9].

Raynaud’s phenomenon represents a vasospastic condition characterized by episodes of digital ischemia triggered by cold exposure or emotional stress, manifesting as digital color changes progressing from white to blue to red [1]. Loss of CGRP-containing nerve fibers in patients with Raynaud’s phenomenon highlights the role of the peptide in maintaining peripheral vasodilation, and pharmacovigilance disproportionality analyses have confirmed associations between anti-CGRP monoclonal antibodies and the development or exacerbation of this condition, consistent with the loss of CGRP-mediated vasodilatory tone in the peripheral circulation [5,103]. A large retrospective cohort study found that initiation of CGRP inhibitors was associated with a modestly increased risk of a composite cardiovascular endpoint, particularly ischemic stroke, although the absolute risk increase was low [93,117].

### 9.5. Sepsis and Multiorgan Dysfunction

While experimental studies indicate that CGRP may exert protective effects in sepsis by modulating inflammatory responses and preserving mitochondrial function, these findings are currently limited to laboratory models [1,5,14]. Studies have found that plasma CGRP levels are elevated in patients with sepsis, and that CGRP can limit the generation of proinflammatory cytokines, including keratinocyte chemoattractant and macrophage inflammatory protein-2, to protect mice against fatal endotoxic shock [1,14]. In experimental models of lipopolysaccharide-induced sepsis-associated cardiomyopathy, administration of CGRP clearly reduced the mortality rate and showed the ability to inhibit lipopolysaccharide-induced sepsis-associated cardiomyopathy in rats [14]. Histological analyses revealed that, compared to the control group, rats in the lipopolysaccharide group exhibited swelling and degeneration of cardiomyocytes, elevated erythrocyte extravasation, and penetration of inflammatory cells, whereas inflammatory responses were reduced in the CGRP-treated group [14].

CGRP is released at an early stage after infection and can then exert antiinflammatory effects on macrophages and other cells to elevate IL-10, among other mechanisms [1,5,14]. Excess proinflammatory mediators such as TNF-α, IL-1, and IL-6 trigger myocardial suppression in sepsis, while compensatory antiinflammatory responses fail to suppress the immune response. Mitochondrial dysfunction is an important factor in the pathogenesis of sepsis and is linked to the prognosis of the condition, and CGRP has been found to positively regulate mitochondrial fusion through stabilization of L-OPA1 [14]. CGRP altered the transcription levels of 96 genes in rat myocardial tissue, including 67 differentially expressed downregulated genes and 29 differentially expressed upregulated genes, with enriched functions related to migraine, innate immune response, protein degradation, RNA metabolism, transcription factors, and signaling pathways [14]. CGRP, as an effector molecule in inflammation, has received increasing attention for its therapeutic potential in sepsis, with studies showing that CGRP plays a role in mitigating microglial activation, suppressing Th1 inflammatory responses, reducing production of TNF-α, IL-2, and IFN-γ, and modulating innate immune responses of macrophages [14].

### 9.6. Neurodegenerative Diseases

CGRP exhibits a dual role in neurodegenerative diseases, acting as a neuroprotective agent under certain circumstances while its dysregulation can contribute to neurotoxicity in others [5,13]. Whereas the neuroprotective effects of CGRP include suppression of inflammation, regulation of intracellular signaling pathways, and promotion of neuronal growth and survival, under pathological conditions, its overexpression or dysregulation is associated with oxidative stress, excitotoxicity, and neuronal death [13]. In the case of Alzheimer’s disease, it has been suggested that exogenous administration of CGRP inhibits tissue infiltration of macrophages and expression of various inflammatory mediators, which in turn attenuates inflammatory responses [13]. The neuroprotective role of CGRP in multiple neuronal populations against neurotoxicity is mediated through pathways whose disruption affects neuronal cell proliferation, differentiation, and survival, thus causing neurodegeneration. CGRP weakens anti-apoptotic pathways and strengthens proliferative pathways in an Akt/ERK-dependent manner, and reduced the mitochondrial toxicity of the apoptosis-inducing toxin N-methyl-4-phenylpyridinium and protected a subpopulation of mesencephalic dopaminergic PC12 cells overexpressing α-synuclein in vitro [13].

**Table 2:** Pathophysiological roles of the CGRP system beyond migraine.

Pathological Area	Role of the CGRP System	Molecular Mechanism Involved	Therapeutic Implication
Oncology (Tumor Immunity) [16,17,116]	Promotes immune evasion and tumor progression.	Preclinical studies suggest that CGRP signaling may contribute to tumor-associated immunosuppression by promoting CD8^+^ T-cell exhaustion, altering dendritic-cell phenotype, and suppressing IL-15 expression in fibroblasts, thereby potentially reducing NK-cell infiltration. However, these findings remain experimental, and the therapeutic value of CGRP blockade has not been established clinically.	Receptor blockade (e.g., with antagonists) has been proposed as a potential combination with immunotherapy to improve anti-tumor surveillance, but this remains preclinical and untested in humans
Oncology (Bone Metastases) [12,16,115]	Promotes proliferation of cancer cells in the bone microenvironment.	Activation of the CLR/p38/HSP27 pathway in metastatic cells.	Use of CGRP antagonists or antibodies to reduce bone metastasis progression.
Chronic pain (chemotherapy-induced neuropathy) [109,110,111]	Contributes to mechanical and cold hypersensitivity.	Upregulation of CLR/RAMP1 in spinal cord; increased calcium-dependent release from sensory neurons.	CGRP receptor antagonists (e.g., BIBN4096) have shown efficacy in preclinical models of neuropathic pain
Neurodegeneration (Alzheimer’s) [5,13,14]	Receptor dysregulation contributes to amyloid pathology.	Inhibition of CGRP signaling improves amyloid pathology through reprogramming of lipid metabolism (HDAC11/LXRβ/ABCA1 pathway).	Repurposing of CGRP antagonists (gepants) as a neuroprotective strategy in Alzheimer’s disease has been suggested by preclinical studies, but remains highly experimental and has not been validated clinically
Sepsis [1,9,14]	Protects against multi-organ dysfunction and sepsis-associated encephalopathy.	Mitigates neuroinflammation and neuronal apoptosis through downregulation of the JNK pathway; preserves mitochondrial function.	Development of stable CGRP analogs for use in intensive care medicine has been proposed based on preclinical models, but no clinical data are available

Recent studies have revealed that CALCRL, a central component of the CGRP receptor, is upregulated in the hippocampus of patients with Alzheimer’s disease dementia and in 5FAD mice, a murine model of Alzheimer’s disease [13]. CGRP antagonists reduce neuroinflammation and Alzheimer’s disease pathology, leading to improvement in learning and memory in young Alzheimer’s disease mice. Inhibition of the CGRP receptor improves Alzheimer’s disease pathology by reprogramming lipid metabolism through HDAC11/LXRβ/ABCA1 signaling, indicating that CGRP antagonists could represent therapeutic strategies for Alzheimer’s disease [13]. The potential role of CGRP in associated synucleinopathies, including Parkinson’s disease, multiple system atrophy, and dementia with Lewy bodies, merits further study given the potentially protective role of CGRP and its related pathways in these conditions. Using an ex vivo intestinal slice culture model from A53T+ mutant mice, CGRP treatment increases enteric α-synuclein accumulation and induces a reactive enteric glial cell phenotype, suggesting that peripheral CGRP signaling may contribute to the gut–brain axis in Parkinson’s disease pathogenesis [24]. A key mechanism in neurodegenerative processes is apoptosis, which is also a major mechanism of neuronal death in Parkinson’s disease, and treatments targeting apoptosis have been considered in synucleinopathies, with CGRP stimulating a proliferative signaling cascade that promotes neuronal survival and increases neurite growth and regeneration [13]. Recent preclinical evidence also suggests that CGRP alleviates epilepsy-related damage through the JAK1-STAT1-P2RX7 signaling axis, reducing seizure frequency and neuroinflammation [118].

### 9.7. Psoriasis and Cutaneous Inflammatory Diseases

CGRP contributes to dermal T-lymphocyte infiltration in psoriasis and is implicated in the pathophysiology of this chronic inflammatory skin disease [1,5]. Its expression is increased across all epidermal layers in psoriatic skin compared to healthy controls, and circulating CGRP levels are elevated and correlate with PASI scores. In vitro, CGRP enhances T-lymphocyte adhesion, migration, and IL-6 secretion, supporting a role in promoting immune cell recruitment to the skin [5].

Psoriatic plaques are characterized by infiltration of activated CD4+ and CD8+ T cells, with CD4+ cells predominantly in the dermis and CD8+ cells in the epidermis [5,119]. CGRP may contribute to disease progression by initiating cutaneous inflammation and promoting keratinocyte proliferation [1,5,119]. Activated keratinocytes amplify this response through the release of chemokines (e.g., CCL20, CXCL1), which recruit T cells and neutrophils, and cytokines (e.g., IL-36, IL-1β), establishing self-sustaining inflammatory loops [1,5,120]. Skin-homing receptors such as CCR4 and cutaneous lymphocyte antigen facilitate T-cell localization to the epidermis, where they produce proinflammatory mediators including IL-17A, further driving keratinocyte activation [1,5,120]. The therapeutic effect of acitretin has been linked, at least in part, to inhibition of CGRP secretion from T lymphocytes [5,119,121].

**Table 3:** CGRP-related pathologies categorized by the strength of clinical and preclinical evidence.

Condition	Evidence Level	Findings	Reference
Migraine (adult)	High (established)	FDA-approved mAbs and gepants	[12]
Pediatric migraine	Moderate (emerging)	Elevated CGRP levels confirmed in children	[122]
Gastrointestinal	Moderate (clinical)	Constipation as AE reveals pro-motility role	[10]
Oncology/metastasis	Low (preclinical)	CGRP promotes bone metastasis in preclinical models via p38 signaling; clinical relevance unknown	[16]
Sepsis	Low (experimental)	Mitochondrial protection and anti-inflammatory effects observed in animal models only	[14]
Alzheimer’s disease	Low (experimental)	Receptor upregulation in murine hippocampal models	[13]

## 10. Therapeutic Pharmacology

### 10.1. CGRP Receptor Antagonists: Gepants

**Figure 5:**
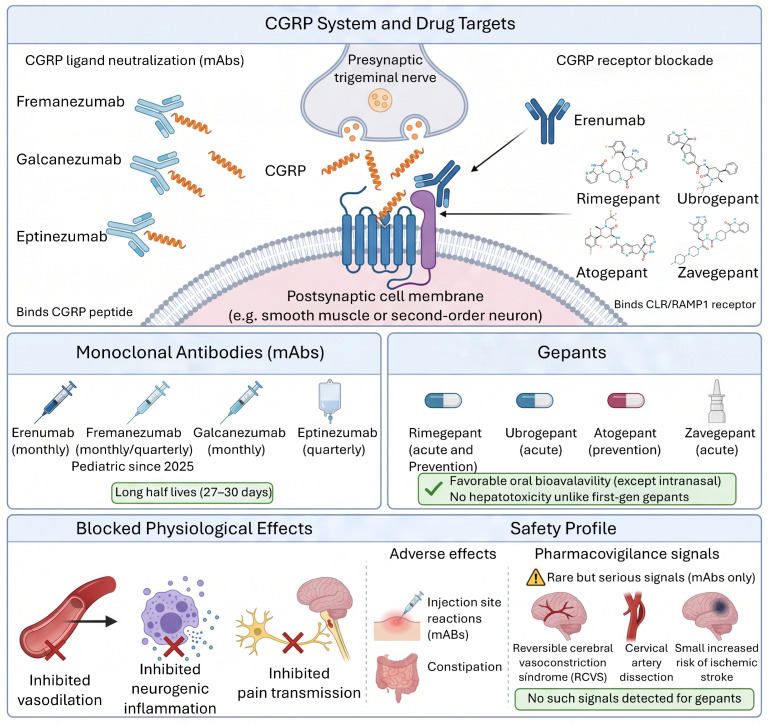
Current landscape of drugs blocking the CGRP system for the treatment of migraine. Two major drug families have been developed to target the CGRP system: gepants (small-molecule CLR-RAMP1 receptor antagonists) and monoclonal antibodies (mAbs) that neutralize the CGRP ligand or block its receptor. Second-generation gepants (rimegepant, ubrogepant, atogepant, zavegepant) exhibit favorable oral bioavailability (except intranasal zavegepant) and lack the hepatotoxicity that limited first-generation compounds (olcegepant, telcagepant). Rimegepant is approved for both acute treatment and prevention of episodic migraine; atogepant is indicated solely for prevention; ubrogepant and zavegepant for acute treatment. Monoclonal antibodies (erenumab, fremanezumab, galcanezumab, eptinezumab) have long half-lives (~27–30 days), enabling monthly or quarterly dosing. Erenumab is the only one that binds the receptor; the others neutralize the CGRP. Fremanezumab was approved in 2025 for pediatric episodic migraine (ages 6–17). Safety profiles are generally favorable, with the most common adverse events being injection site reactions (mAbs) and constipation (especially with atogepant and erenumab). Pharmacovigilance analyses have identified rare but serious signals of reversible cerebral vasoconstriction syndrome (RCVS) and cervical artery dissection associated with some mAbs, as well as a small increased risk of ischemic stroke in observational studies; no such signals have been detected for gepants. Detailed knowledge of these pharmacological differences enables personalized treatment selection based on patient comorbidities and risk profiles.

Small-molecule CGRP receptor antagonists (gepants) comprise a pharmacological class that has evolved across successive generations with improvements in oral bioavailability, efficacy, and safety profile [11]. Development began with olcegepant (BIBN4096BS), which demonstrated efficacy in acute migraine but required intravenous administration due to low oral bioavailability [12]. These early studies established that CGRP receptor blockade could relieve migraine without the vasoconstrictive effects associated with triptans [11] (Figure 5). Telcagepant was the first orally available gepant with demonstrated efficacy in both acute and preventive settings, but its development was discontinued due to hepatotoxicity associated with sustained use [12].

Second-generation gepants, including rimegepant, ubrogepant, atogepant, and zavegepant, show improved pharmacokinetic profiles and no clinically relevant hepatotoxicity signals [12]. Rimegepant is approved for both acute treatment and prevention of episodic migraine [123]. Ubrogepant is indicated for acute treatment, whereas atogepant is used for prevention [12,123]. Zavegepant, administered intranasally, provides an alternative route with rapid systemic absorption and avoids first-pass hepatic metabolism [12].

Across pivotal trials, these agents consistently demonstrate modest but clinically meaningful efficacy. For acute treatment, pain freedom at two hours is achieved in approximately 20–24% of patients, compared with 12–15% with placebo, with numbers needed to treat around 10–14 [43]. Preventive regimens reduce monthly migraine days by approximately 3.5–4.5 days over 12 weeks, compared with 2.5–3.5 days with placebo [124]. Intranasal zavegepant shows a comparable efficacy profile with rapid onset, while dysgeusia is the most common adverse event associated with this formulation [12,43].

Combination strategies have also been explored. The TANDEM study indicates that concomitant use of ubrogepant for acute treatment and atogepant for prevention does not introduce new safety signals, supporting the feasibility of dual gepant therapy [123]. Furthermore, real-world evidence supports the use of dual preventive therapy with two CGRP antagonists (e.g., a monoclonal antibody and a gepant) in patients with chronic migraine who have exhausted other options, showing significant reductions in headache days without major safety concerns [125,126].

### 10.2. Monoclonal Antibodies Against CGRP and Its Receptor

Monoclonal antibodies targeting the CGRP system represent an alternative therapeutic approach to gepants, with four agents currently approved, including three antibodies that bind the CGRP and one that targets the receptor [103]. These therapies are characterized by long half-lives, enabling infrequent dosing schedules, and by a mechanism of action that does not involve vasoconstriction, supporting their use in patients with cardiovascular risk.

Erenumab is a fully human monoclonal antibody that binds to the CGRP receptor, specifically targeting the calcitonin receptor-like receptor in complex with RAMP1, thereby preventing activation by both CGRP-α and CGRP-β [103]. It is administered as a monthly subcutaneous injection at doses of 70 or 140 mg. Clinical trials have shown that erenumab reduces monthly migraine days by approximately 3–4 days in episodic migraine and produces larger reductions in chronic migraine, with a dose-dependent effect [103].

In contrast, fremanezumab, galcanezumab, and eptinezumab act by directly binding the CGRP and preventing its interaction with the receptor [103]. Fremanezumab exhibits similar affinity for CGRP-α and CGRP-β and can be administered either monthly (225 mg) or quarterly (675 mg), with comparable efficacy between regimens [103]. This flexible dosing strategy distinguishes fremanezumab from other agents in the class. In addition to evidence from randomized trials, real-world studies have demonstrated sustained effectiveness. The pan-European PEARL study of fremanezumab reported that 58.5% of patients achieved a ≥50% reduction in monthly migraine days over 6–12 months [127]. Similarly, the TRIUMPH study of galcanezumab showed responder rates of 63.1% at 3 months, although with different response criteria for episodic and chronic migraine [128]. Long-term observational studies, including the EUREkA cohort, have demonstrated sustained reductions in monthly headache days over 24 months, with baseline factors such as depression, obesity, and migraine with aura negatively affecting treatment persistence [19]. Similarly, the German Pain e-Registry reported significantly higher 6-month persistence for CGRP monoclonal antibodies (89.4%) compared to conventional oral preventives (34–43%) [129,130]. Notably, fremanezumab became in 2025 the first anti-CGRP therapy approved for pediatric episodic migraine, extending its clinical applicability. A recent meta-analysis confirmed that blood CGRP levels are significantly elevated during both ictal and interictal periods in children and adolescents with migraine compared to controls, supporting CGRP as a potential diagnostic biomarker in this population [122]. Moreover, case series have reported the effectiveness and tolerability of anti-CGRP monoclonal antibodies in pediatric patients with intractable chronic daily headaches [131]. While most CGRP-targeted therapies are currently registered for adults, the elevated CGRP levels observed in pediatric migraineurs suggest a potential role for CGRP as a diagnostic biomarker. However, the clinical use of anti-CGRP therapies in children and adolescents remains limited to the approved indication for fremanezumab in episodic migraine (ages 6–17), and further controlled trials are needed before broader pediatric use can be recommended [122].

Galcanezumab also binds CGRP, with preferential affinity for the α isoform, and is administered monthly following an initial loading dose. Clinical trials have demonstrated consistent reductions in monthly migraine days across both episodic and chronic migraine populations, with efficacy comparable to other ligand-targeting antibodies [103].

Eptinezumab differs from other agents in that it is administered by quarterly intravenous infusion, resulting in immediate systemic bioavailability. This pharmacological feature is associated with a more rapid onset of action, with some studies reporting reductions in migraine burden from the first day after administration. Its efficacy in both episodic and chronic migraine is comparable in magnitude to that observed with subcutaneously administered antibodies [103].

Across clinical trials, anti-CGRP monoclonal antibodies consistently reduce monthly migraine days by approximately 3–5 days in episodic migraine and 4–8 days in chronic migraine, compared with smaller reductions observed with placebo [103]. Beyond migraine, galcanezumab (300 mg) and eptinezumab (400 mg) have demonstrated efficacy in reducing attack frequency in episodic cluster headache, although their benefit in the chronic form remains less definitive [132,133]. A comparative profile of approved drugs targeting the CGRP system, including gepants and monoclonal antibodies, is provided in Table 4.

### 10.3. Comparative Efficacy

Indirect comparisons among monoclonal antibodies targeting CGRP or its receptor consistently indicate broadly comparable efficacy across agents, although no direct head-to-head trials are available [134]. A network meta-analysis found that fremanezumab 225 mg ranked highest in both efficacy and safety among anti-CGRP monoclonal antibodies for episodic migraine, while galcanezumab 240 mg showed the highest efficacy for reducing monthly migraine days [135]. Another meta-analysis including 8926 patients from 18 randomized clinical trials showed that eptinezumab, galcanezumab, fremanezumab, and erenumab produced similar reductions in monthly migraine days. Consistent with these findings, Bucher indirect comparisons assessing the 75% response rate at 12 weeks revealed no statistically significant differences among eptinezumab 300 mg, erenumab 140 mg, fremanezumab 675 mg quarterly, and galcanezumab 240 mg monthly in chronic migraine [136].

Comparative ranking analyses provide a more nuanced view of relative efficacy. A meta-analysis including five anti-CGRP agents (erenumab, fremanezumab, galcanezumab, eptinezumab, and rimegepant) found that fremanezumab 225 mg monthly, erenumab 140 mg monthly, and atogepant 60 mg daily ranked highest across multiple efficacy outcomes based on the surface under the cumulative ranking curve [137]. However, differences between treatments were modest, and all agents showed clear superiority over placebo.

Across trials, clinically meaningful response rates are consistently observed. In episodic migraine, 50% response rates range from 43% to 62% with monoclonal antibodies compared with 23% to 27% with placebo, whereas in chronic migraine they range from 27% to 41% versus 15% to 18%, respectively. These effects translate into numbers needed to treat of approximately 4–6 in episodic migraine and 6–9 in chronic migraine [103].

Subgroup analyses further support the consistency of treatment effects. Studies with gepants such as ubrogepant and atogepant have shown similar efficacy in men and women, despite the predominance of female participants in clinical trials [12].

### 10.4. Safety and Tolerability

The safety profile of gepants is characterized by favorable tolerability, with adverse events predominantly mild to moderate. The most commonly reported adverse events include nausea, somnolence, and fatigue, with incidences generally comparable to placebo [123]. Gastrointestinal adverse events have been consistently identified across pharmacovigilance analyses, with atogepant showing a higher frequency of signals compared to rimegepant. Constipation is particularly notable with atogepant during preventive use, occurring in approximately 6–11% of patients versus 2% with placebo [123]. Hepatobiliary safety remains a relevant consideration given the hepatotoxicity observed with first-generation gepants; however, currently approved agents show improved hepatic safety profiles. In clinical trials, elevations of alanine or aspartate aminotransferase greater than three times the upper limit of normal were rare and reversible upon treatment discontinuation [138]. These pharmacokinetic considerations may influence treatment selection in patients receiving multiple concomitant medications [123].

Monoclonal antibodies targeting CGRP or its receptor are associated with low rates of systemic adverse events, consistent with their target specificity and limited tissue distribution [103]. The most frequent adverse events are injection site reactions, reported in 3–17% of patients, and typically manifesting as transient erythema, pruritus, or induration. Additionally, cases of delayed urticaria (12–48 h post-injection) have been reported, suggesting a non-IgE-mediated hypersensitivity mechanism. These reactions can often be managed with H1-antihistamine premedication, allowing patients to safely continue effective prophylaxis [139]. Constipation has also been reported, particularly with erenumab, with incidences of approximately 3–5% compared with 1–2% under placebo [134]. A systematic review of spontaneous reporting system data confirmed consistent signals for injection site reactions, alopecia, and constipation, and identified rare but serious signals for reversible cerebral vasoconstriction syndrome (RCVS) and cervical artery dissection associated with certain monoclonal antibodies, whereas no such signals were detected for gepants [21]. In patients with relevant comorbidities, observational data from the SAFE-CGRP study indicate that treatment-related worsening is uncommon, occurring in a small proportion of cases and generally resolving after discontinuation [6].

Theoretical cardiovascular concerns arise from the physiological role of CGRP as a vasodilatory and cardioprotective peptide [5]. Pooled analyses of randomized trials with erenumab found no increase in major adverse cardiovascular events compared with placebo, although high-risk patients were generally excluded [140]. Nevertheless, clinicians have warned against an unacceptable vasoconstrictive risk of CGRP-related therapies specifically in patients with Moyamoya angiopathy [141]. In any case, a retrospective cohort study reported a small but statistically significant increase in cardiovascular events among CGRP inhibitor users, driven largely by ischemic stroke, although the absolute risk was low [93]. Pharmacovigilance analyses have also detected signals for palpitations and, for monoclonal antibodies, coronary artery dissection and Prinzmetal angina [22]. Observational data in patients with hypertension suggest that clinically relevant worsening of blood pressure is infrequent [6]. Disproportionality analyses have identified associations with Raynaud’s phenomenon, consistent with the role of CGRP in peripheral vasodilation, although the absolute risk appears low [103]. Ambulatory blood pressure monitoring studies have not demonstrated significant changes in systolic or diastolic pressure, although monitoring remains advisable in patients with pre-existing cardiovascular disease [94].

### 10.5. Clinical Considerations and Therapeutic Positioning

Expert consensus guidelines position gepants as effective options for the acute pharmacological treatment of migraine, consistent with recommendations from the American Headache Society and the International Headache Society [123]. They are particularly relevant for patients who do not respond adequately to triptans, experience incomplete relief, or present contraindications or intolerance to these agents. Their use is limited in specific clinical contexts, including severe renal impairment (creatinine clearance < 15 mL/min) and severe hepatic dysfunction (Child–Pugh C), while caution is warranted in moderate hepatic impairment [123]. As substrates of cytochrome P450 3A4 and P-glycoprotein, gepants require careful consideration of drug–drug interactions, particularly with strong inhibitors or inducers of these pathways; co-administration with agents such as ketoconazole or clarithromycin may necessitate dose adjustment or avoidance depending on the specific compound [123].

In contrast, monoclonal antibodies targeting CGRP or its receptor are indicated for the preventive treatment of migraine and represent the first class of therapies developed on the basis of disease-specific mechanisms [12]. Switching between different anti-CGRP monoclonal antibodies has emerged as a viable strategy for patients who do not respond adequately to the first agent. Real-world studies indicate that approximately one-third of non-responders may achieve a meaningful response after switching, particularly when the second antibody targets a different mechanism of action (e.g., from an anti-receptor to an anti-ligand antibody) [103,142].

Preventive treatment is generally considered in patients with four or more migraine days per month, although thresholds vary across guidelines [12]. Compared with traditional prophylactic agents, monoclonal antibodies offer greater target specificity and a reduced burden of systemic adverse effects, as well as monthly or quarterly dosing schedules that may improve adherence [103], although monitoring may be advisable in patients with relevant cardiovascular comorbidities. Clinical studies in patients with prior failure of multiple preventive therapies indicate that approximately 30% of these individuals achieve a meaningful response. Furthermore, anti-CGRP monoclonal antibodies have proven effective in patients with medication-overuse headache (MOH) without requiring prior drug withdrawal, significantly reducing monthly migraine days and acute medication intake [96,143,144]. The optimal duration of treatment remains to be defined, although available data suggest that 20–30% of responders may maintain clinical benefit after discontinuation following 6–12 months of therapy [124]. Additionally, routine screening for obstructive sleep apnea (OSA) is recommended before initiating anti-CGRP monoclonal antibodies in chronic migraine, as managing this underdiagnosed comorbidity can reduce migraine-related disability [145]. However, a meta-analysis found that after discontinuation, monthly migraine days increased significantly compared to during treatment, although they remained lower than pretreatment levels, indicating that the disease-modifying potential of these therapies is limited [124].

## 11. Conclusions and Future Perspectives

CGRP has become one of the most investigated neuropeptides in recent decades, and represents an example of how basic research in molecular biology can be translated into therapeutic innovations [11]. Structural characterization of the CLR-RAMP1-Gs protein complex by cryo-electron microscopy has provided insight into the molecular mechanisms by which the peptide activates its receptor and triggers intracellular signaling cascades, revealing details of the peptide–receptor interactions that inform the design of agonists, antagonists, and allosteric modulators with improved pharmacological properties [15]. Elucidation of the signaling pathways activated downstream of the CGRP receptor has revealed considerable complexity, with the peptide capable of activating not only the canonical cAMP-PKA pathway, but also MAPK cascades, PI3K-Akt pathways, and calcium-dependent signaling through coupling to different G proteins depending on cellular context [5,13].

Recognition of the physiological functions of CGRP has expanded considerably beyond its initial role as a vasodilator, revealing its involvement in processes such as bone metabolism regulation, promotion of wound healing and tissue regeneration, modulation of immune responses, and regulation of energy metabolism [5,13]. Recent studies demonstrating that CGRP-expressing sensory neurons promote tissue healing through modulation of myeloid cell infiltrates and promotion of angiogenesis suggest that the CGRP system could represent a target for enhancing tissue repair in conditions where this process is compromised [17]. The dual and seemingly paradoxical role of CGRP in conditions such as osteoarthritis, where the peptide simultaneously contributes to cartilage degradation and protection of subchondral bone, illustrates the complexity of CGRP system functions and underscores the need for therapeutic approaches that consider these potentially divergent effects in different tissue compartments [5].

The development of gepants and monoclonal antibodies targeting the CGRP system represents one of the most important therapeutic advances in neurology in recent decades, providing patients with migraine with specific treatment options that lack the vasoconstrictor effects and usage limitations associated with triptans [12]. Efficacy data for these agents show that approximately 50 percent of patients with episodic migraine achieve at least a 50 percent reduction in monthly migraine days when receiving preventive treatment with monoclonal antibodies, and that approximately 20 percent of patients treated acutely with gepants achieve pain freedom at two hours [12,25,103]. However, these data also show that a significant proportion of patients do not respond adequately to these therapies, suggesting heterogeneity in migraine pathophysiology and the possible existence of subtypes in which mechanisms other than CGRP play a predominant role [12]. Recent real-world analyses have introduced the concept of ‘false nonresponders’, patients who do not meet the 50% monthly migraine day reduction criterion but still experience meaningful improvements in pain intensity, medication use, disability, or quality of life. These patients may benefit from continued treatment beyond 6 months [146]. The identification of predictive biomarkers of response to CGRP-targeted therapies represents a research priority that could enable personalized treatment and more rational selection of candidates for these costly therapies [12,102]. Moreover, recognizing that a subset of patients remains refractory to CGRP blockade, recent research has validated the pituitary adenylate cyclase-activating polypeptide (PACAP) as a parallel and independent pathway in migraine pathophysiology. Results from recent clinical trials, such as the HOPE trial, have positioned anti-PACAP monoclonal antibodies as the most promising clinical alternative or complement for patients who do not respond to anti-CGRP therapies, marking a new era in migraine neuropharmacology [147,148].

Theoretical cardiovascular safety concerns with blockade of the CGRP system, based on the well-documented cardioprotective and vasodilatory role of endogenous CGRP, have not been confirmed by accumulated data from clinical trials and observational studies, although continued vigilance is recommended, particularly in patients with established cardiovascular disease [5,9]. The SAFE-CGRP study demonstrated that blockade of the CGRP system in patients with cardiovascular and autoimmune comorbidities was associated with treatment-related worsening in only 2.9% of cases [6]. However, results from the IMMUNO-CGRP study indicate that patients with comorbid autoimmune diseases have a significantly lower likelihood (OR 0.61) of achieving a 50% response rate compared to controls, despite maintaining a comparable safety profile [149].

Raynaud’s phenomenon and exacerbation of pre-existing hypertension represent adverse events that require monitoring, reflecting the interruption of physiological CGRP-mediated vasodilation [12,25,103]. Constipation observed with both gepants and monoclonal antibodies confirms the physiological importance of CGRP in gastrointestinal motility and represents a consideration in patient selection and adverse event management [10,134].

Future perspectives for CGRP research are broad and include several directions that could expand both fundamental understanding and therapeutic applications of the CGRP system. The development of selective CGRP receptor agonists with improved metabolic stability could have applications in conditions where enhancement of CGRP signaling would be therapeutically beneficial, including refractory hypertension, peripheral vascular disease, promotion of wound healing, and prevention of osteoporosis [5,9,17]. Preclinical studies demonstrating beneficial effects of lipidated CGRP analogs with plasma half-lives exceeding 10 h in models of wound healing and muscle regeneration suggest the feasibility of this approach [5]. The development of selective CGRP receptor antagonists with central nervous system penetration could have applications in conditions where central CGRP signaling contributes to pathology, although this approach would require careful characterization of effects on physiological functions of central CGRP.

Characterization of allosteric variants of the CGRP receptor and identification of alternative binding sites distinct from the orthosteric site could enable the development of positive or negative allosteric modulators with distinct pharmacological properties, including subtype selectivity if CLR heteromers or functional splicing variants of the receptor are identified [13]. Research on the role of the CGRP system in neurodegenerative diseases is at a relatively early stage, but suggests a complex situation, with evidence of both neuroprotective and potentially deleterious effects depending on context and disease stage. Studies showing that inhibition of the CGRP receptor improves Alzheimer’s disease pathology in murine models through reprogramming of lipid metabolism suggest that CGRP antagonists could have applications beyond migraine, although translation to humans requires careful validation [13]. The identification of the role of the CGRP system in meningeal lymphatic vessels and its contribution to cerebrospinal fluid clearance opens new research directions into mechanisms by which CGRP could influence cerebral fluid homeostasis and contribute to neurological conditions where lymphatic drainage is compromised [13].

Preclinical models suggest that sensory nerve-derived CGRP may promotes bone metastasis progression through activation of the p38/HSP27 pathway in cancer cells. However, the clinical significance of this axis remains to be validated in human trials [5,13,16]. CGRP signaling has also been implicated in immune evasion in oral squamous cell carcinoma [116] and in promoting a dense extracellular matrix in breast cancer, further supporting the rationale for repurposing CGRP antagonists as adjuvant immunotherapies. Studies showing that ablation of CGRP or blockade of its receptor reduces tumor-associated angiogenesis and tumor growth in preclinical models suggest potential applications as adjuvant treatment in anticancer therapy, particularly in solid tumors where angiogenesis is important for progression [16]. Evaluation of combinations of CGRP-targeted therapies with other established treatments, including combinations of gepants with different pharmacokinetic profiles for simultaneous acute and preventive treatment, as demonstrated in the TANDEM study, could optimize therapeutic outcomes in patients with difficult-to-treat migraine [12]. Novel pharmacological strategies include the design of dual-target ligands (e.g., compound PCC0105005) that concurrently antagonize CGRP receptors and activate serotonin 5-HT1F receptors, aiming for synergistic efficacy with limited central nervous system side effects [150]. Investigation of mechanisms of resistance or loss of response to CGRP-targeted therapies, observed in approximately 20 to 30 percent of initially responding patients, represents a priority for maintaining long-term therapeutic benefit [12,124].

Finally, here, we integrate current knowledge of the CGRP system from the molecular to the clinical level, demonstrating that the biological complexity of this neuropeptide, encompassing alternative gene processing, post-translational modifications essential for receptor engagement, a heterotrimeric CLR-RAMP1 receptor architecture revealed at atomic resolution, and convergent intracellular signaling through cAMP-PKA, MAPK, and calcium pathways, explains both its pleiotropic physiological roles and its causal involvement in migraine and a broad spectrum of systemic diseases. The clinical validation of CGRP-targeted therapies across three generations of gepants and four approved monoclonal antibodies exemplifies how structural and mechanistic insights can drive rational drug development. Emerging evidence implicating the CGRP system in bone metastasis progression, meningeal lymphatic dysfunction, Alzheimer’s disease pathology, and immunosuppression of the tumor microenvironment positions CGRP research at the intersection of neuroscience, oncology, and immunology, with therapeutic implications that extend well beyond the current migraine indication.

## Figures and Tables

**Table 4:** Comparative profile of approved drugs targeting the CGRP system.

Feature	Rimegepant	Ubrogepant	Atogepant	Zavegepant	Erenumab	Fremanezumab	Galcanezumab	Eptinezumab
Class	Gepant (receptor antagonist)	Gepant (receptor antagonist)	Gepant (receptor antagonist)	Gepant (receptor antagonist)	Monoclonal antibody (anti-receptor)	Monoclonal antibody (anti-peptide)	Monoclonal antibody (anti-peptide)	Monoclonal antibody (anti-peptide)
Target	CLR/RAMP1	CLR/RAMP1	CLR/RAMP1	CLR/RAMP1	CLR/RAMP1 receptor	CGRP-α and β	CGRP-α and β (pref. α)	CGRP-α and β
Indication(s)	Acute and Preventive	Acute	Preventive	Acute	Preventive	Preventive (adults and pediatric)	Preventive	Preventive
Route of admin.	Oral	Oral	Oral	Intranasal	Subcutaneous	Subcutaneous	Subcutaneous	Intravenous
Dose (approx.)	75 mg	50–100 mg	10–60 mg/day	10 mg	70–140 mg/month	225 mg/month or 675 mg/quarter	240 mg (loading dose), then 120 mg/month	100–300 mg/quarter
Half-life	~11 h	5–7 h	~11 h	6.5 h	~28 days	~30 days	~27 days	~27 days
Common adverse events	Nausea, fatigue	Nausea, somnolence	Constipation, nausea	Dysgeusia	Injection site reactions, constipation	Injection site reactions	Injection site reactions	Infusion reactions
Based on [12,21,103,123]								

## Data Availability

No new data were created or analyzed in this study. Data sharing is not applicable to this article.

## Abbreviations

The following abbreviations are used in this manuscript:

ACE	Angiotensin-converting enzyme
Akt	Protein kinase B/Akt
ATF4	Activating transcription factor 4
ATP	Adenosine triphosphate
CALCA	Calcitonin related polypeptide alpha gene
CALCB	Calcitonin related polypeptide beta gene
cAMP	Cyclic adenosine monophosphate
CBP	CREB-binding protein
CCL10	C-C motif chemokine ligand 10
CCL17	C-C motif chemokine ligand 17
CCL22	C-C motif chemokine ligand 22
CD206	Cluster of differentiation 206
CGRP	Calcitonin gene-related peptide
CGRP1	CGRP receptor type 1
CGRP-α	Alpha isoform of calcitonin gene-related peptide
CGRP-β	Beta isoform of calcitonin gene-related peptide
CLR	Calcitonin receptor-like receptor
CNS	Central nervous system
CREB	cAMP response element-binding protein
CSD	Cortical spreading depression
CXCL9	C-X-C motif chemokine ligand 9
DAG	Diacylglycerol
ECE-1	Endothelin-converting enzyme 1
eNOS	Endothelial nitric oxide synthase
Epac	Exchange protein directly activated by cAMP
ERK	Extracellular signal-regulated kinase
ERK1/2	Extracellular signal-regulated kinases 1 and 2
FAK	Focal adhesion kinase
FDA	Food and Drug Administration
GABA	Gamma-aminobutyric acid
GM-CSF	Granulocyte-macrophage colony-stimulating factor
GRK	G protein-coupled receptor kinase
Gs	Stimulatory G protein
HSP27	Heat shock protein 27
IDE	Insulin-degrading enzyme
IFN-γ	Interferon gamma
IL-1ß	Interleukin-1 beta
IL5	Interleukin 5
IL-6	Interleukin-6
IP3	Inositol trisphosphate
KATP	ATP-sensitive potassium channel
Ly6C	Lymphocyte antigen 6 complex, locus C
M2	Alternatively activated macrophage phenotype
MAPK	Mitogen-activated protein kinase
MEK	MAPK/ERK kinase
Mrgprb2	Mas-related G protein-coupled receptor member B2 (mouse)
MRGPRX2	Human homolog of Mrgprb2
mRNA	Messenger RNA
NADPH	Nicotinamide adenine dinucleotide phosphate
NF-κB	Nuclear factor kappa B
OPG	Osteoprotegerin
PACAP	Pituitary adenylate cyclase-activating polypeptide
PASI	Psoriasis area and severity index
PC1	Prohormone convertase 1
PC2	Prohormone convertase 2
PI3K	Phosphatidylinositol 3-kinase
PKA	Protein kinase A
PKC	Protein kinase C
Raf	Rapidly accelerated fibrosarcoma kinase
RAMP1	Receptor activity-modifying protein 1
RANKL	Receptor activator of nuclear factor kappa-B ligand
Rap1	Ras-related protein 1
RCP	Receptor component protein
RCVS	Reversible cerebral vasoconstriction syndrome
RNA	Ribonucleic acid
Rp-cAMPS	Rp-adenosine 3′,5′-cyclic monophosphorothioate
Runx2	Runt-related transcription factor 2
SAX	Acylated CGRP analog
SNARE	Soluble N-ethylmaleimide-sensitive factor attachment protein receptor
SOD3	Superoxide dismutase 3
Src	Src family tyrosine kinase
SRp55	Serine/arginine-rich splicing factor 55
Th2	T helper type 2
TNF-α	Tumor necrosis factor alpha
TRPM3	Transient receptor potential melastatin 3
TRPV1	Transient receptor potential vanilloid 1
VE-cadherin	Vascular endothelial cadherin
